# Hippo Stabilises Its Adaptor Salvador by Antagonising the HECT Ubiquitin Ligase Herc4

**DOI:** 10.1371/journal.pone.0131113

**Published:** 2015-06-30

**Authors:** Birgit L. Aerne, Ieva Gailite, David Sims, Nicolas Tapon

**Affiliations:** 1 Apoptosis and Proliferation Control Laboratory, The Francis Crick Institute, Lincoln’s Inn Fields Laboratory, 44 Lincoln’s Inn Fields, London, United Kingdom; 2 The Breakthrough Toby Robins Breast Cancer Research Centre, Institute of Cancer Research, Chester Beatty Laboratories, London, United Kingdom; University of Dayton, UNITED STATES

## Abstract

Signalling through the Hippo (Hpo) pathway involves a kinase cascade, which leads to the phosphorylation and inactivation of the pro-growth transcriptional co-activator Yorkie (Yki). Despite the identification of a large number of pathway members and modulators, our understanding of the molecular events that lead to activation of Hpo and the downstream kinase Warts (Wts) remain incomplete. Recently, targeted degradation of several Hpo pathway components has been demonstrated as a means of regulating pathway activity. In particular, the stability of scaffold protein Salvador (Sav), which is believed to promote Hpo/Wts association, is crucially dependent on its binding partner Hpo. In a cell-based RNAi screen for ubiquitin regulators involved in Sav stability, we identify the HECT domain protein Herc4 (HECT and RLD domain containing E3 ligase) as a Sav E3 ligase. Herc4 expression promotes Sav ubiquitylation and degradation, while Herc4 depletion stabilises Sav. Interestingly, Hpo reduces Sav/Herc4 interaction in a kinase-dependent manner. This suggests the existence of a positive feedback loop, where Hpo stabilises its own positive regulator by antagonising Herc4-mediated degradation of Sav.

## Introduction

The Hippo (Hpo) pathway has emerged as a major regulator of growth and tissue size [1,2]. It was originally identified in *Drosophila* using genetic screens for mutations resulting in tissue overgrowth [3–12]. The core of the Hpo pathway in *Drosophila* comprises two kinases, Hpo and Warts (Wts). Activated Hpo phosphorylates the downstream kinase Wts, which leads to the Wts-mediated phosphorylation of the transcriptional co-activator Yorkie (Yki) [7,13]. Phosphorylated Yki is sequestered in the cytosol by 14-3-3 proteins, thereby preventing activation of Yki target genes [14,15]. In the nucleus, Yki associates with a number of transcription factors, such as the TEA (Transcription enhancer factor) domain-containing protein Scalloped (Sd) [16,17], to induce the expression of growth-promoting genes (e.g. *myc*, *bantam* and *diap1)* [13,18–20]. Hence, inactivation of Yki by Wts inhibits growth through the transcriptional repression of these pro-growth regulators. The Hpo pathway is highly conserved between mammals and *Drosophila* [1,2]. In humans, the Hpo orthologues MST1/2 (Mammalian Sterile 20-like) phosphorylate the Wts orthologues LATS1/2 (Large Tumor Suppressor), leading to the phosphorylation of the Yki orthologues YAP and TAZ [21–25].

Over the last decade, various genetic screens and proteomic approaches have identified many new components of the pathway [26]. This has generated a vast amount of information and increased understanding of the molecular mechanisms involved in the regulation of this pathway, leading to the notion that Hpo signalling acts as a sensor for mechanical cues, apico-basal and planar polarity and G-protein coupled receptors [1,2,21,27,28]. Thus, Hpo signalling is a key effector of tissue architecture in growth control.

However, despite the ever-expanding complexity of Hpo upstream signalling, the regulation and function of the core components still remain incompletely understood. In particular, apart from phosphorylation, the role of covalent protein modifications, such as ubiquitylation and SUMOylation has been little explored. One well-characterised instance is the regulation of the Yki orthologs YAP and TAZ by the SCF^Slimb/β-TrCP^ E3 ligase complex [29,30]. Sequential phosphorylation of a YAP/TAZ phospho-degron by Casein Kinase 1∂/ε and LATS1/2 leads to recognition and degradation by SCF^Slimb/β-TrCP^. Moreover, several other components of the Hpo pathway have been reported to be targets of ubiquitin-dependent proteasomal degradation, such as RASSF1A, RASSF5/NORE1, Mob1 and Wts/LATS1 [31–36].

The present study focuses on the tumor suppressor protein Salvador (Sav), which, together with Hpo and Wts, was one of the first Hpo pathway components to be identified [5,6]. Sav is a WW domain-containing scaffold protein, which interacts with the Hpo kinase via their C-terminal SARAH (Salvador, RASSF, Hippo) domains [5,6,37]. The functional importance of Sav/Hpo dimerisation for Hpo kinase activity remains unclear, since Hpo/MST dimerisation is sufficient to promote activation by trans-phosphorylation [38–40]. However, genetic evidence in flies and vertebrates clearly indicates that Sav is required for Hpo pathway activity. For instance, loss of fly *sav* phenocopies the overgrowth phenotype of *hpo* mutants [5,6,41,42], while knockout mice for WW45/Sav1 display YAP-dependent phenotypes in multiple tissues [9,41–43]. Recent work indicates that Sav promotes membrane recruitment of Hpo, where it can phosphorylate and activate Wts [44]. Another clear consequence of Sav/Hpo dimerization is the phosphorylation and stabilization of Sav, both in flies and mammals [7,9,45,46]. However, it is not clear whether Sav phosphorylation by Hpo is responsible for its stabilization, since a point mutant in the ATP-binding pocket (Lysine to Arginine, K/R) of Hpo/MST still stabilizes Sav [9,45]. Here, we use an RNAi screening approach to identify regulators of Sav stability. We identify the HECT E3 ubiquitin ligase Herc4 as a negative modulator of Hpo signalling. In addition, we show that Hpo can stabilise Sav by interfering with Herc4-dependent Sav degradation.

## Material and Methods

### *Drosophila* cell culture and expression constructs

*Drosophila* S2 cells were transfected with Effectene transfection reagent (Qiagen) and grown in *Drosophila* Schneider’s medium (Life Technologies) containing 10% FBS (Sigma), 50μg/ml penicillin and 50 μg/ml streptomycin. Expression plasmids were generated using Gateway® technology (Life Technologies). Open reading frames (ORF) were PCR amplified from existing plasmids or cDNA (DGRC, https://dgrc.cgb.indiana.edu/vectors/) and cloned into Entry vectors (pENTR^TM^/D-TOPO). Vectors from the Drosophila Gateway Vector Collection (http://www.ciwemb.edu/labs/murphy/Gateway%20vectors.html) were used as Destination vectors. All expression plasmids were sequence-verified. Point mutations were generated using the Quikchange® Multi Site-Directed Mutagenesis Kit (Stratagene).

### dsRNA production and treatment

RNAi library generation work-plan can be found in the supplementary figures (S2–S4 Figs). dsRNAs were synthesized using the Megascript T7 kit (Ambion). DNA templates for dsRNA synthesis were PCR-amplified from genomic DNA using primers that contained the 5’ T7 RNA polymerase-binding site sequence. dsRNA primers were designed using the DKFZ RNAi design tool (http://www.dkfz.de/signaling2/e-rnai/)

RNAi screening was carried out as outlined in S3 Fig. Follow-up RNAi experiments were carried out in 6 well plates using S2 cells. 1-2x10^6^ cells were plated per well and cells left to settle. After 3 h the medium was removed and replaced with 1 ml of serum free Schneider’s medium containing dsRNAs (20 μg). Cells were soaked for 30 min and then 2 ml of full Schneider’s medium was added. Transfections were carried out 24 h after dsRNA treatment.

### Immunoprecipitation and immunoblot analysis

For immunoprecipitation assays, cells were lysed in Lysis Buffer (50 mM Tris pH8, 150 mM NaCl, 1% NP-40, and 1 mM EGTA) supplemented with 0.1M NaF, phosphatase inhibitor cocktails 2 and 3 (Sigma) and protease inhibitor cocktail (Roche). Cell extracts were cleared at 16,000 *g* for 20 min at 4°C. FLAG-tagged proteins were purified using anti-FLAG M2 Affinity Agarose Gel (Sigma), whereas immunoprecipitation of Myc-tagged proteins was carried out using Protein A Sepharose (Sigma). Detection of purified proteins and associated complexes was performed by immunoblot analysis using chemiluminescence (Pierce). Western Blots were probed with mouse anti-FLAG (M2, Sigma), rabbit anti-FLAG (Sigma), mouse anti-Myc (9E10, Santa Cruz Biotechnology), rabbit anti-Myc (Santa Cruz Biotechnology), rat anti-HA (3F10, Roche Applied Science), mouse anti-Tubulin (E7, Developmental Studies Hybridoma Bank, DSHB) and mouse anti-GFP (in-house produced), rat anti-Yki [47], and rabbit [48] and guinea pig anti Sav (a kind gift from G. Halder).

### Ubiquitylation assays

For ubiquitylation assays S2 cells were transfected with Myc-Sav and HA-Ubiquitin and, unless otherwise stated, treated with proteasome inhibitors for 4 h. Cells were harvested and lysed in Lysis buffer (see above). Lysates were immunoprecipitated with an anti-Myc antibody and Protein A Sepharose (Sigma), and subjected to western blot analysis. Ubiquitylation was detected using an antibody against HA-Ubiquitin. Lysis buffer containing 5mM N-Ethylmaleimide (NEM) (Sigma) was used to block deubiquitylating enzyme activity.

### Proteasome inhibitor treatment

For proteasome inhibition experiments, cells were treated with 50μM MG132 (Calbiochem) and 50μM calpain inhibitor I (Ac-LLnL-CHO or LLnL) (Sigma) for 4 h before cell lysis. Ubiquitylation assays were carried out after proteasome inhibitor treatment, unless stated otherwise.

### Western blot analysis of fly heads

Heads were cut off from frozen animals and lysed in RIPA buffer (10 mM Tris HCl pH 7.5, 150 mM NaCl, 1% (v/v) Triton X-100, 0.1% (w/v) SDS, 1% (w/v) Sodium deoxycholate) supplemented with 0.1M NaF, phosphatase inhibitor cocktails 2 and 3 (Sigma) and protease inhibitor cocktail (Roche). Tissue lysates were incubated on ice for 10 min and subsequently cleared by centrifugation at 16,000 *g* for 10 min at 4^°^C. 150ug of total lysate was loaded per lane.

### Immunofluorescence

Wing imaginal discs from third-instar larvae were dissected, fixed for 20 min in 4% formaldehyde, washed three times in PBS supplemented with 0.1% Triton X-100 (PBS-T) and pre-blocked in PBS supplemented with 0.3% Triton X-100 and 10% normal goat serum (NGS) for 1 h. Discs were incubated overnight in the primary antibody diluted in PBS-T containing 10% NGS, followed by three washes with PBS-T, and incubation with a secondary antibody in PBS-T/10% NGS for 2 h at room temperature. After three further washes, discs were mounted in Vectashield mounting medium with DAPI (Vectorlabs). Rat anti-Ci^155^ antibody (Developmental Studies Hybridoma Bank, monoclonal 2A1) was used at 1:20. Secondary anti-rat Alexa 647 antibody (Molecular Probes) was used at 1:200. Fluorescence images were acquired on a Zeiss LSM710 confocal laser scanning microscope (40x objective lens).

### Sequence Alignments

Multiple sequence alignment and phylogenetic tree analysis was carried out using Clustal Omega (EMBL-EBI). Protein sequence accession numbers were as follows: Itch *hs* BAB39389.1 (GenBank), Nedd4 *hs* AAI44285 (AAI44285), Smurf1 *hs* EAL23885 (EAL23885), Herc1 *hs* Q15751.2 (UniProtKB/Swiss-Prot), Herc2 *hs* O95714.2 (UniProtKB/Swiss-Prot), Herc3 *hs* Q15034.1 (UniProtKB/Swiss-Prot), Herc4 *hs* Q5GLZ8.1 (UniProtKB/Swiss-Prot), Herc5 *hs* Q9UII4.2 (UniProtKB/Swiss-Prot), Herc6 *hs* Q8IVU3.2 (UniProtKB/Swiss-Prot), Herc4 *dm* (CG9153) Q5GLZ8.1 (UniProtKB/Swiss-Prot).

### Generation of *herc4* CRISPR mutant

Introduction of a frame-shift mutation in the *herc4* gene was performed using the CRISPR/Cas9 system [49]. Suitable target sites for guide RNAs (sgRNA) were determined using http://www.flyrnai.org/crispr/. Sense and anti-sense oligos of the selected sgRNA were annealed, phosphorylated and cloned into the expression vector pCFD3 according to the protocol described on http://www.crisprflydesign.org/grna-expression-vectors/. DNA was injected into embryos of *y* ^*1*^, *M{nos-Cas9*.P}ZH-2A w* transgenic flies (Bloomington stock number 54591) by the Fly Facility at the University of Cambridge Department of Genetics. G0 flies were then crossed with the *w;;TM3/TM6* balancer strain; subsequently, G1 male flies were crossed with the same balancer strain, followed by genomic DNA screening for frame-shift mutations. We also balanced a wild type progeny of the injected founders to use as a control strain (*herc4* ^*ctrl*^).

### Analysis of *Drosophila* adult wings

Adult wings were mounted using Euparal mounting medium (Agar Scientific) and imaged using a Zeiss Axio-plan 2 imaging system, using Optovar optics (1.6x) and a 2.5x objective (Plan-NEOFLUAR, Zeiss) connected to a Leica DFC420C camera. Wings were subsequently measured and quantified using ImageJ.

### Genotypes

Fig 6: left panels: *w; ubi-GFP*::*sa*v/+; *hh-GAL4/+*, right panels: *w; ubi-GFP*::*sav/+; hh-GAL4/UAS-Herc4*.

Fig 10: *w; UAS-CD8-GFP; rn-GAL4*. *w; UAS-herc4; rn-GAL4*., *w; UAS-herc4-C/A; rn-GAL4*. *w; UAS-CD8-GFP; rn-GAL4*, *UAS-sav*. *w; UAS-herc4; rn-GAL4*, *UAS-sav*. *w; UAS-herc4-C/A; rn-GAL4*, *UAS-sav*. *w; UAS-CD8-GFP; rn-GAL4*, *UAS-sav-S413A*. *w; UAS-sav-S413A; rn-GAL4*, *UAS-herc4*. *w; UAS-herc4-C/A; rn-GAL4*, *UAS-sav-S413A*.

Fig 11: *w;; rn-GAL4/ctrl*. *w;; rn-GAL4*, *UAS-sav/ctrl*. *w;; rn-GAL4*, *UAS-sav-S413A/ctrl*. *w;; rn-GAL4/herc4* ^*C6*.*3*^. *w;; rn-GAL4*, *UAS-sav/ herc4* ^*C6*.*3*^. *w;; rn-GAL4*, *UAS-sav-S413A/ herc4* ^*C6*.*3*^.

## Results

### Sav is ubiquitylated and degraded by the proteasome

**Fig 1:**
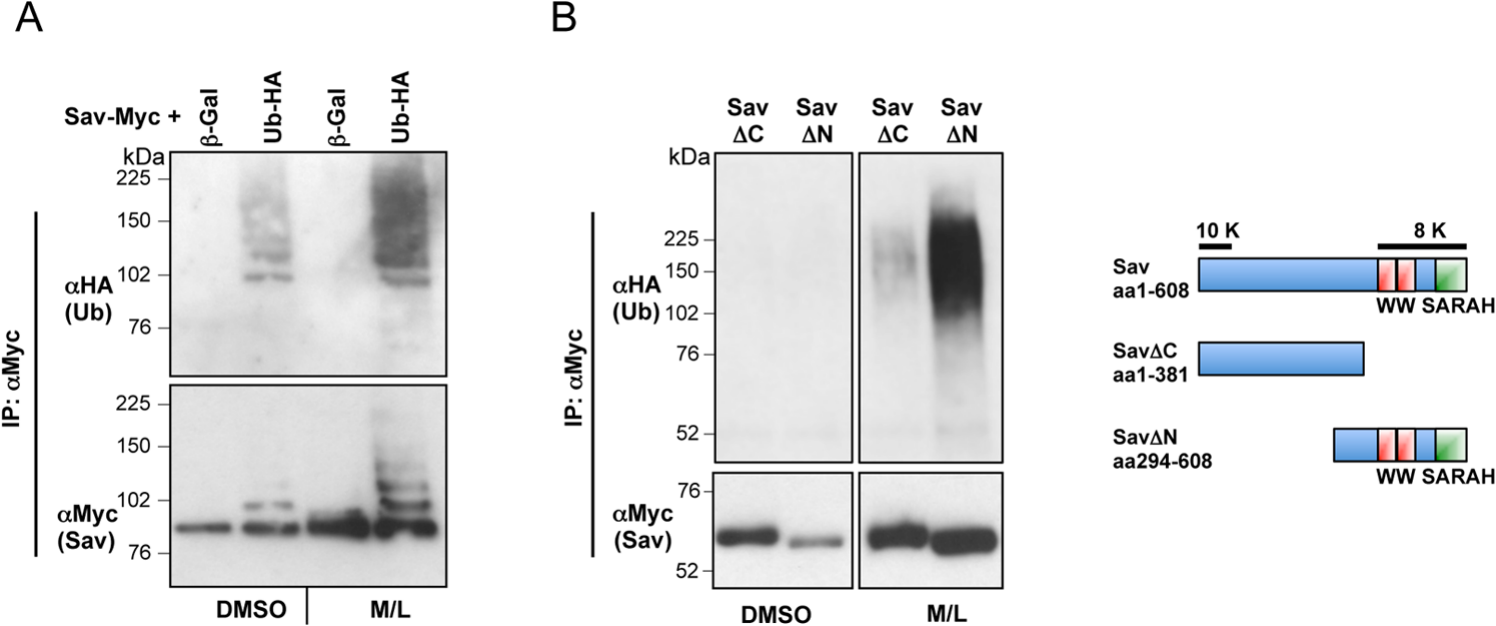
Sav is ubiquitylated predominantly in the C-term half of the protein. (A) Sav is stabilised and shows increased ubiquitylation in the presence of proteasome inhibitors (MG132 and LLnL). Myc-tagged Sav was expressed in S2 cells with either Ub-HA or ß-Galactosidase (ß-Gal) in the presence of proteasome inhibitors (MG132 and LLnL, M/L) or DMSO control. Sav ubiquitylation assay shows incorporation of HA-tagged ubiquitin. (B) Left panel: Sav ubiquitylation occurs in the C-terminal half of the protein. Sav deletions (∆C and ∆N) were tested for ubiquitylation as described above. Right panel: schematic representation of Sav protein structure and distribution of Lysine residues. Sav contains eighteen Lysine residues (K), which could serve as potential ubiquitylation sites: ten are located near the N-terminal end of the protein, whereas the remaining eight are located in the C-terminus.

Sav is an unstable protein and its stability is regulated by the proteasome system (Fig 1*A*, [7,9,45,46]). As proteasomal degradation is triggered by poly-ubiquitylation of target proteins, we performed a cell-based ubiquitylation assay by co-expressing Myc-tagged Sav with HA-tagged ubiquitin, followed by Myc immunoprecipitation (Fig 1*A*). This revealed the presence of Sav poly-ubiquitylated species, which were stabilised by treatment with proteasome inhibitors. Ubiquitin conjugation primarily occurs through the formation of an isopeptide bond between the ubiquitin C-terminus and the epsilon amino group of lysine residues on the substrate [50]. Sav contains eighteen lysine residues, clustered at both termini of the protein, ten are contained within amino acids 12 to 57 and eight are located in the area containing the conserved WW and SARAH domains (Fig 1*B*). To establish the domain where Sav ubiquitylation occurs, we analysed Sav truncations spanning either the N or C terminal part of the protein (Sav∆C and Sav∆N). As shown in Fig 1*B*, Sav ubiquitylation occurs within the conserved C-terminal domain, comprising the WW and SARAH domain.

### Hpo stabilises Sav by inhibiting its ubiquitylation

**Fig 2:**
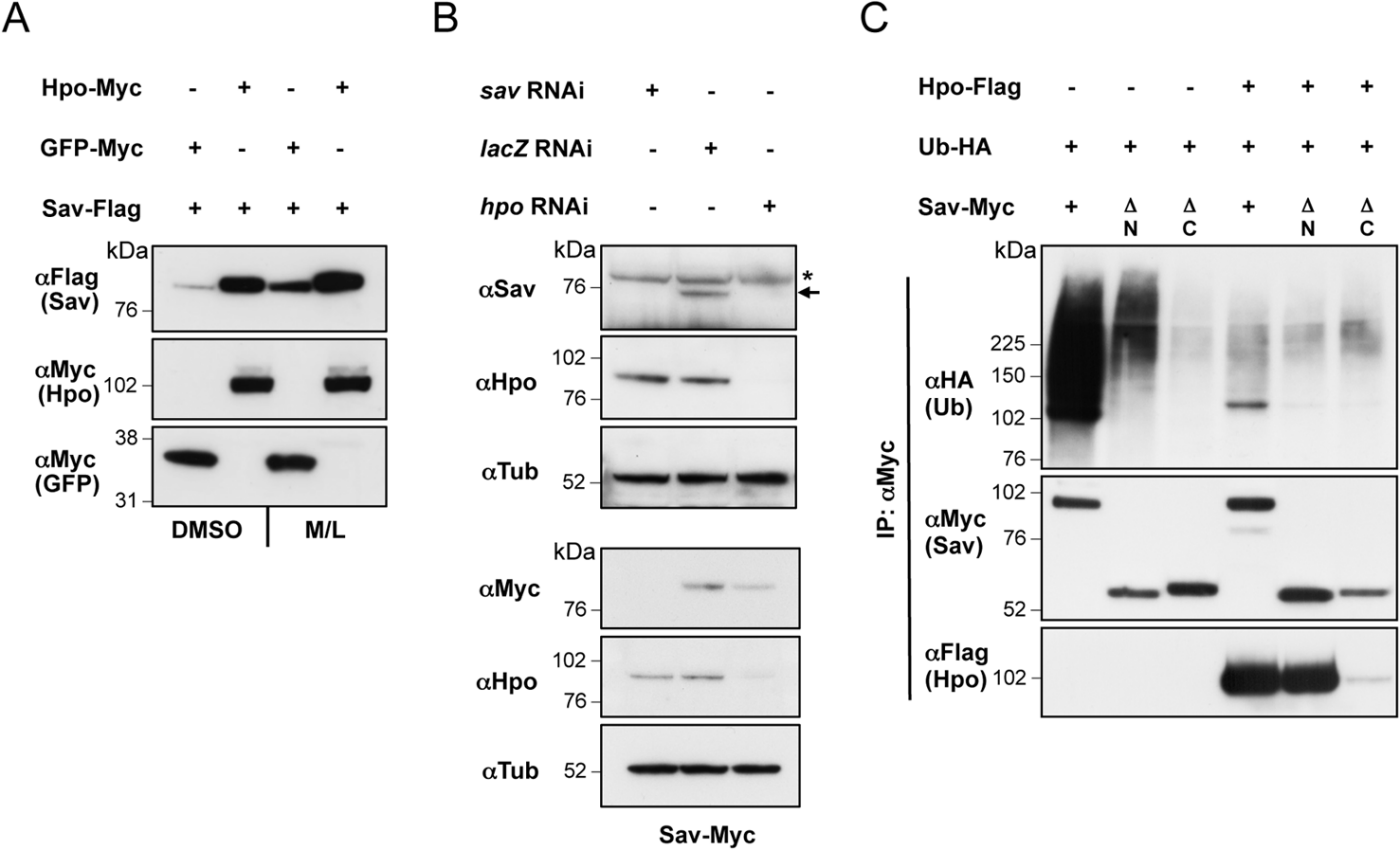
Hpo stabilises Sav by inhibiting Sav ubiquitylation. (A) Sav is stabilised when co-expressed with Hpo. Baseline Sav expression is increased upon proteasome inhibitor treatment (M/L). (B) *hpo* depletion by RNAi destabilises Sav protein levels. Top panel: endogenous Sav levels (arrow) in non-transfected S2 cells. Asterisk indicates a non-specific band detected by the anti-Sav antibody. Bottom panel: levels of transfected Myc-tagged Sav. Sav depletion by RNAi is used as a control. (C) Hpo inhibits ubiquitylation of Sav. Sav, Sav∆N, and Sav∆C were analysed for ubiquitylation in the presence or absence of co-expressed Hpo. Hpo prevents ubiquitylation of full-length Sav and Sav∆N, while Sav∆C is not ubiquitylated. (A-C) S2 cells were transfected with the indicated plasmids or treated with the indicated dsRNAs prior to lysis. Lysates were processed for western blot analysis or used in co-IP.

We and others have observed that exogenous Sav is stabilised by Hpo co-expression, while Hpo depletion causes endogenous Sav downregulation (Fig 2*A* and 2*B*, [7,9,45,46,48]). In order to further investigate the role of Hpo in regulating Sav stability, we carried out ubiquitylation assays using full-length Sav or Sav truncations (Sav∆C and Sav∆N) in the presence or absence of Hpo. Co-expression of Hpo resulted in a reduction in Sav ubiquitylated species (Fig 2*C*), indicating that Hpo regulates Sav stability by preventing its ubiquitylation.

**Fig 3:**
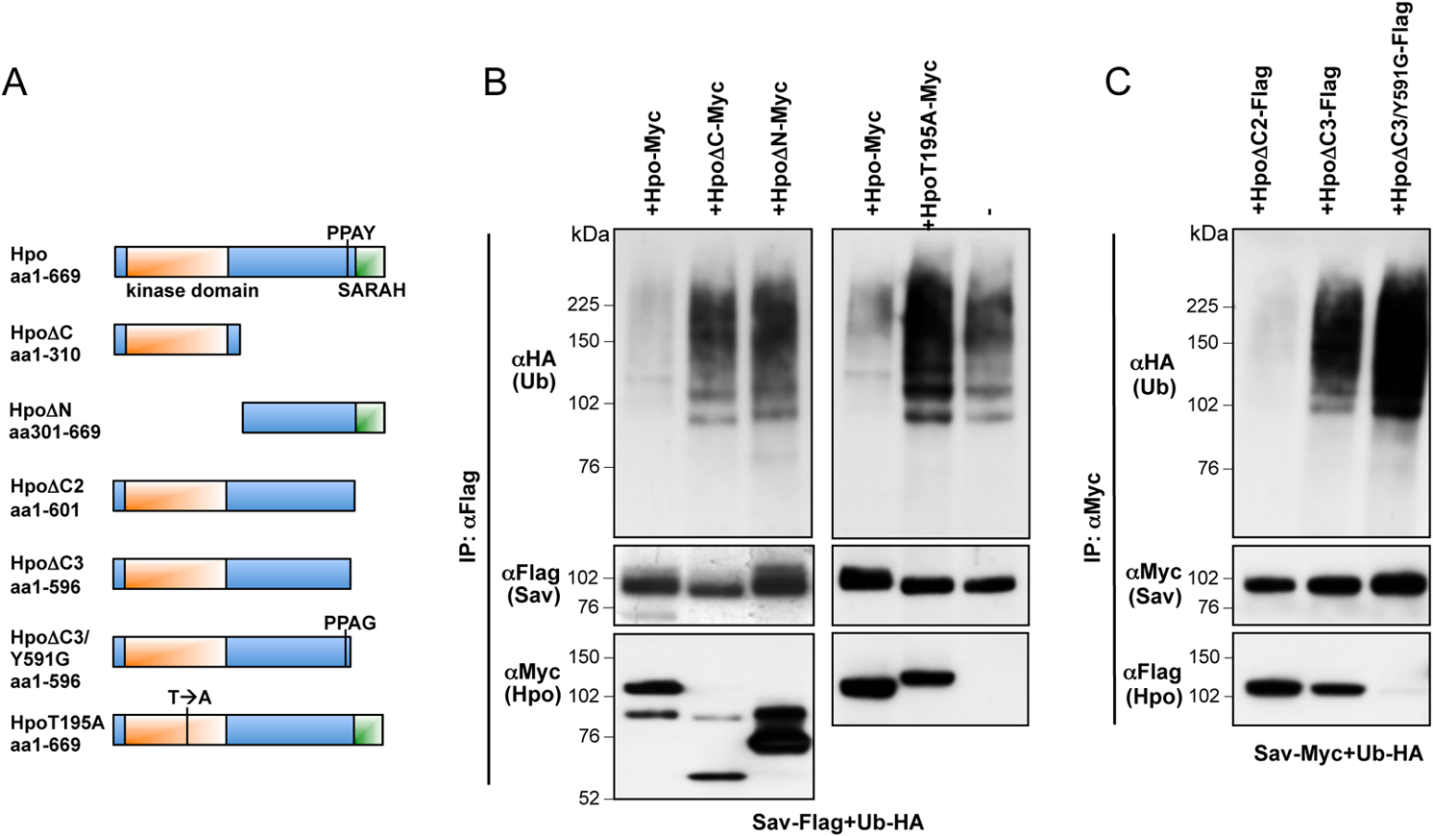
Binding of active Hpo to Sav is required to inhibit Sav ubiquitylation. **(**A) Schematic representation of Hpo constructs used. Protein domains and point mutations in the Hpo T-loop inactivating Hpo kinase (HpoT195A) and PPxY motif (Y591G) are indicated. (B) Sav ubiquitylation assays with co-expressed Hpo truncations (Hpo∆N and ∆C) or Hpo kinase dead (HpoT195A). While full-length Hpo prevents Sav ubiquitylation, truncations lacking the kinase domain or C-terminus, or a kinase-dead Hpo have no effect. (C) Hpo C-terminal deletions (Hpo∆C2 and Hpo∆C3) and a Hpo C-terminal deletion mutated for the PPxY motif (Hpo∆C3/Y591G) affect Sav ubiquitylation proportionally to their ability to bind Sav (compare upper and lower panels).

Since Sav ubiquitylation occurs in the C-terminal domain responsible for Hpo binding, Hpo could sterically interfere with ubiquitin ligase binding or ubiquitin transfer. Alternatively, since Hpo is known to phosphorylate Sav ([7,9,45,46,51] and this report), phosphorylation might prevent ligase binding or ubiquitylation. To investigate these possible mechanisms, we analysed the effect of several Hpo truncations and mutants on Sav ubiquitylation (Fig 3*A*). Hpo constructs that bind but fail to phosphorylate Sav, either by deletion of the kinase domain (Hpo∆N) or point mutation in the T-loop domain (at residue 195 –HpoT195A), were unable to prevent Sav ubiquitylation (Fig 3*B*). Similarly, we tested two C-terminal truncations of Hpo lacking the SARAH domain but still containing the Hpo PPxY motif, the longer of which still binds Hpo robustly (Hpo∆C2), while the shorter truncation is reduced in its ability to bind Hpo (Hpo∆C3), as well as a version of Hpo∆C3 carrying a mutation in the PPxY motif (Hpo∆C3/Y591G) motif, which cannot bind Sav at all (Fig 3*C*). These experiments showed that the strength of Hpo/Sav binding correlates with the effect of Hpo on Sav ubiquitylation, with Hpo∆C2 strongly blocking Sav ubiquitylation, and Hpo∆C3 and Hpo∆C3/Y591G having increasingly weaker effects (Fig 3*C*). These results indicate that Hpo requires both Sav binding and kinase activity to inhibit Sav ubiquitylation, thereby excluding steric interference as the sole mechanism for Hpo action.

### A ubiquitin ligase RNAi screen identifies Herc4 as a Sav E3 ligase

**Fig 4:**
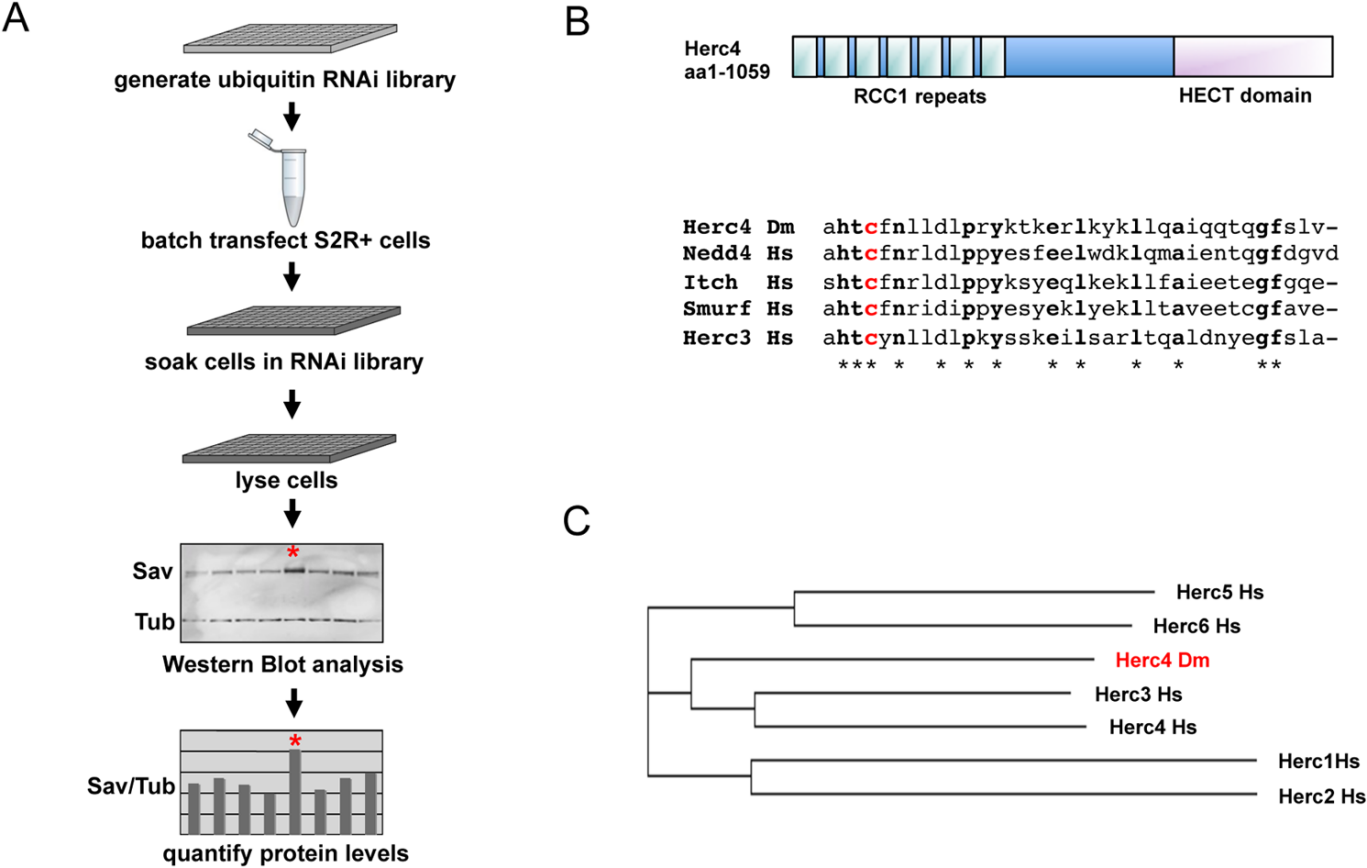
Ubiquitin ligase RNAi screen identifies Herc4 as a Sav E3 ligase. **(**A) E3 RNAi screen work-plan **(**B) Diagram depicting the Herc4 protein domain structure and alignment of HECT domains. Herc4 contains seven RCC1 repeats and a HECT E3 ligase domain. Protein alignment shows the last 36 aa of *Drosophila melanogaster* Herc4 aligned with the corresponding sequences of human Nedd4, Itch, Smurf, and Herc3. The conserved catalytic cysteine is shown in red. (C) Phylogenetic tree of human Herc1-6 and *Drosophila melanogaster* Herc4.

To identify proteins responsible for Sav ubiquitylation, we carried out a cell-based RNAi screen using a *Drosophila* dsRNA sub-library containing known and candidate genes involved in ubiquitylation (378 genes involved in ubiquitin conjugation and ubiquitin binding). The effect of dsRNAs on Sav-Myc expression was analyzed by standard Western-blot analysis. Using this approach we identified the HECT domain protein Herc4 (CG9153) as a Sav E3 ligase (Fig 4*A*). *Drosophila* Herc4 contains a RLD (RCC1-like domains) domain comprising 7 RCC1-like repeats and a HECT domain [52] (Fig 4*B*). RLD domains are structural scaffolds with a β-propeller architecture believed to be involved in protein-protein interactions, whereas the HECT domain is a conserved E3 ligase domain [53,54]. HECT domains bind specific ubiquitin-conjugating enzymes (E2s), accept ubiquitin from the E2 to form a ubiquitin-thioester intermediate with the HECT active cysteine (Fig 4*B*), then transfer ubiquitin to the substrate. Herc4 belongs to the subfamily of small Herc E3 ligases, which in humans comprises 4 members, Herc3-6 [52]. In *Drosophila* there are only 2 small Herc E3 ligases, Herc2 and Herc4. *Drosophila* Herc4 is marginally more related to human Herc4 than human Herc3 (45% and 44% identity between CG9153 and hsHerc4 and hsHerc3, respectively; Fig 4*C*).

### Herc4 controls Sav stability and ubiquitylation *in vitro* and *in vivo*

**Fig 5:**
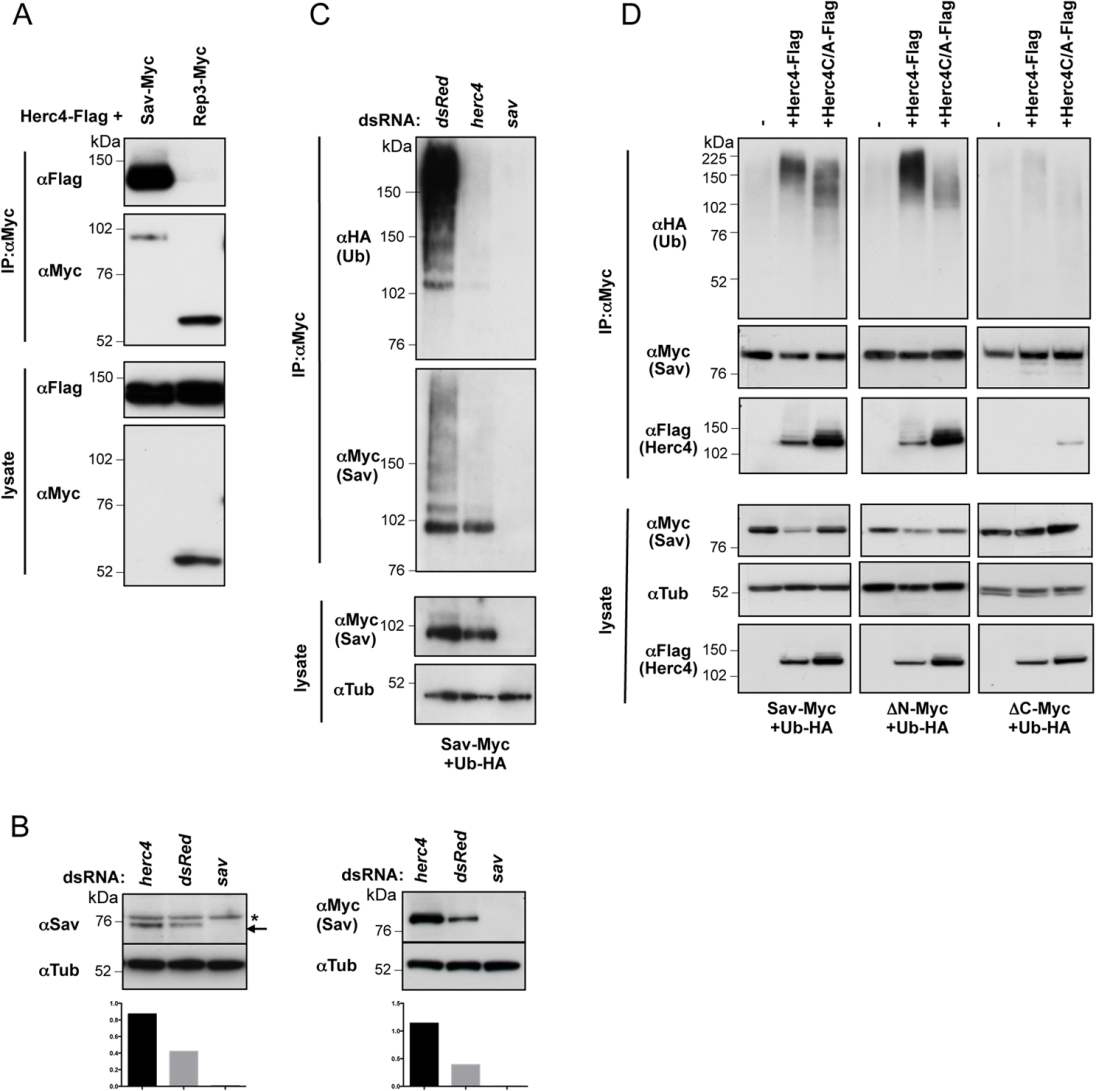
Herc4 controls Sav stability and ubiquitylation. (A) Sav binds to Herc4. Myc-tagged Sav was immunoprecipitated using a Myc antibody and bound Herc4-Flag protein detected using Flag antibody. Myc-tagged *Drosophila* Rep3 was used as control. (B) *herc4* RNAi stabilises Sav protein levels. Left panel: endogenous Sav levels (arrow) in non-transfected S2 cells. Asterisk indicates a non-specific band detected by the anti-Sav antibody. Left bottom panel: quantification presented as ratio of Sav/Tub levels. Right panel: levels of transfected Myc-tagged Sav. Right bottom panel: quantification presented as ratio of Sav-Myc/Tub levels (C) *herc4* RNAi reduces Sav ubiquitylation. Sav ubiquitylation assay of Myc-tagged Sav expressed in S2 cells, treated with *dsRed* RNAi, *herc4* RNAi or *sav* RNAi. Ubiquitylation assay was performed in the presence of 5mM NEM to block deubiquitylating enzyme activity (D) Overexpression of Herc4 results in destabilisation and increased ubiquitylation of Sav, which is dependent on the active site cysteine. Sav ubiquitylation assay of Myc-tagged Sav, Sav∆N and Sav∆C co-expressing either Flag-Herc4 or Flag-Herc4C/A.

HECT E3 ligases physically interact with their substrates [54]. To determine whether Herc4 associates with Sav, we performed co-immunoprecipitation (co-IP) experiments in S2 cells using tagged Herc4 and Sav. Herc4 robustly associated with Sav, but not with a control protein, Rep3 (Fig 5*A*). We identified Herc4 based on the ability of *herc4* RNAi to increase Sav protein levels in S2 cells (Fig 5*B*). To further investigate the functional significance of this finding, the role of Herc4 in Sav ubiquitylation was analysed. We performed a cell-based ubiquitylation assay in the presence of the deubiquitylating enzyme inhibitor NEM to preserve ubiquitylated species. As shown in Fig 5*C*, *herc4* RNAi resulted in a marked reduction of Sav ubiquitylation compared to control RNAi treatment, indicating that Herc4 is required for Sav ubiquitylation.

To assess the specificity of Herc4 as a Sav E3 ligase, we analyzed the effect of Herc4 overexpression on Sav ubiquitylation. Expression of Herc4 enhanced Sav ubiquitylation and reduced Sav protein levels in cell lysates, while a catalytically impaired Cysteine to Alanine mutant (Herc4C/A) had a reduced effect (Fig 5*D*). In agreement with our previous observations (Fig 1*B*), the C-terminal portion of Sav (Sav∆N), but not the N-terminal (Sav∆C), binds to and is ubiquitylated by Herc4 (Fig 5*D*). In contrast, two other HECT domain E3 ligases, Su(dx) and Nedd4, did not promote Sav ubiquitylation (S1 Fig).

**Fig 6:**
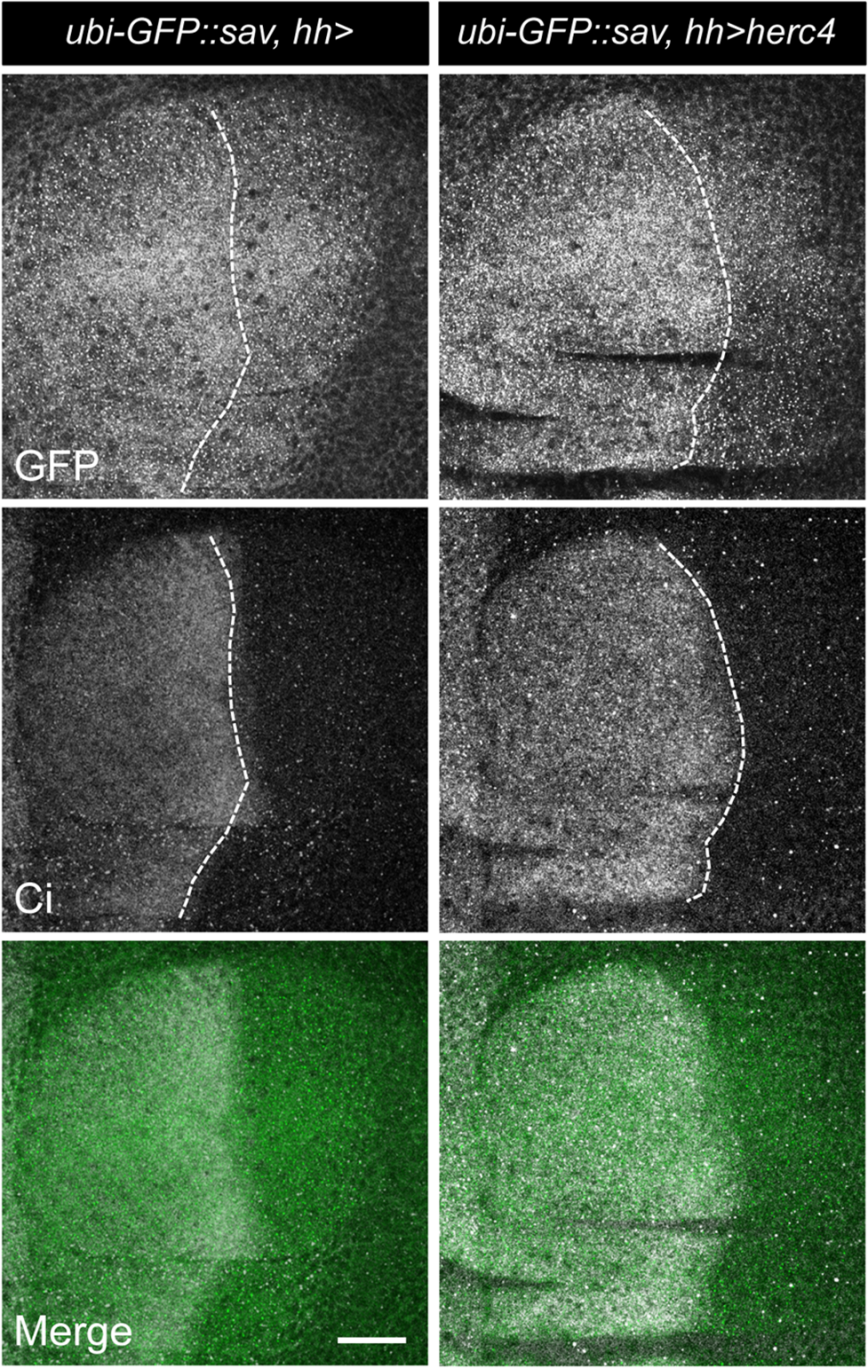
Herc4 overexpression reduces Sav protein levels *in vivo*. GFP-tagged Sav was expressed under the control of the *ubiquitin* promoter (*ubi-GFP*::*Sav*) and analysed in 3^rd^ instar larval wing discs expressing *hh-GAL4* alone (left panel) or *UAS-herc4* in the posterior compartment (*hh>herc4*). White dotted lines denote the border between the anterior and posterior compartments of the wing. Anti-Ci staining marks the anterior compartment. Scale bar = 100μm.

Next, we explored the effect of Herc4 on Sav stability *in vivo*. We generated a GFP-tagged *sav* transgene, expressed under the control of the ubiquitin promoter (*ubi-GFP*::*sav*). Using the GAL4/UAS system, *UAS-Herc4* was expressed in the posterior compartment of wing imaginal discs, the larval precursor to the adult wing, using the *hh-Gal4* (referred to as *hh>herc4*) driver. Cubitus interruptus (Ci) staining was used to differentiate the anterior (Ci-positive) and posterior (Ci-negative) compartments of the wing imaginal disc. Herc4 overexpression caused depletion of Sav levels (Fig 6, right panels), indicated by the reduction in the GFP signal. These results are consistent with our biochemical data showing that Herc4 promotes Sav ubiquitylation and proteasomal degradation.

### Mapping the Herc4/Sav interaction domains

**Fig 7:**
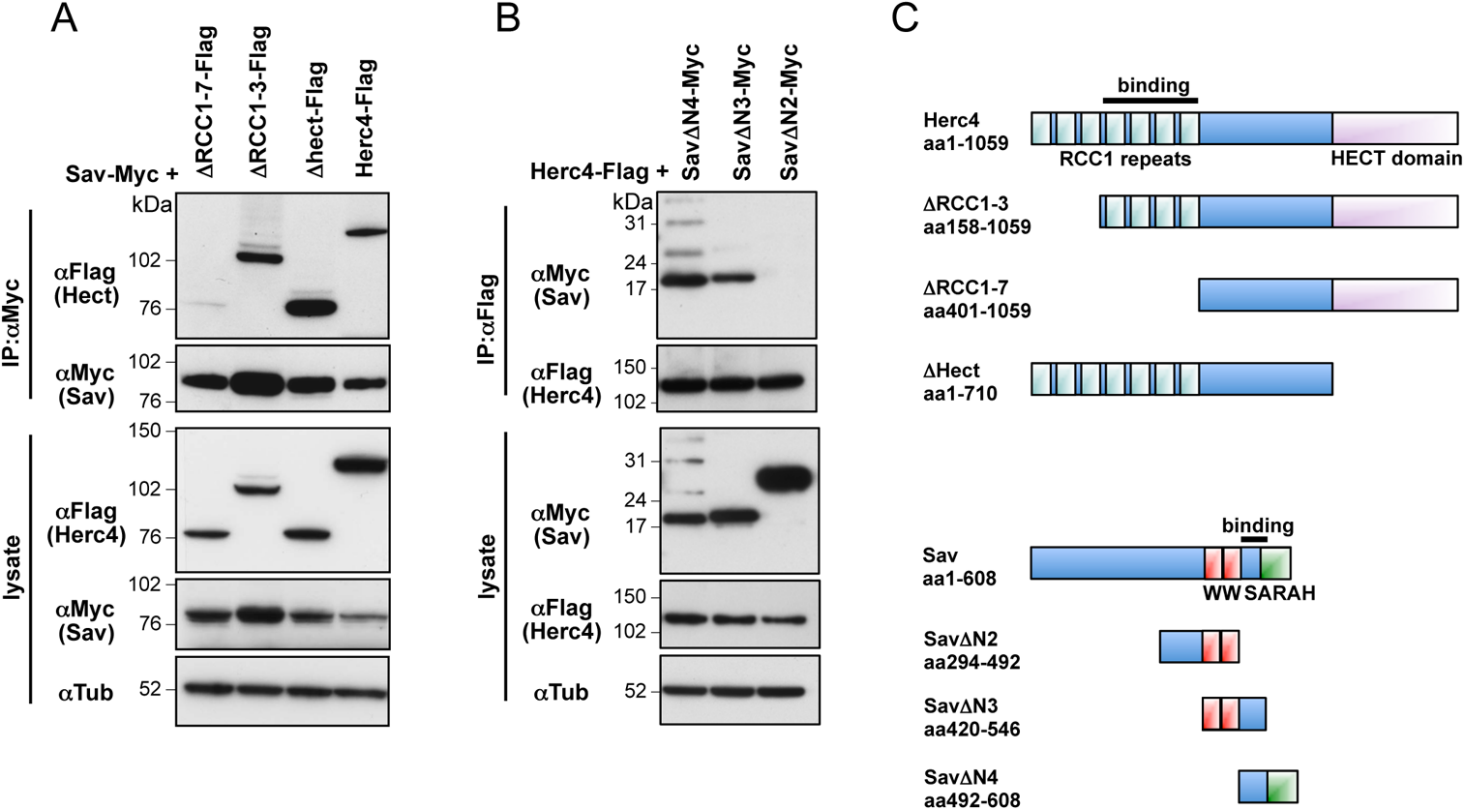
Mapping of Herc4/Sav binding. **(**A) Mapping of Herc4 interaction domain with Sav. Co-IPs of Sav with Herc4 truncations lacking the N-terminal RCC1 domains (∆RCC1-3, ∆RCC1-7) or the HECT domain (∆HECT). The RCC4-7 repeats are required for binding of Herc4 to Sav. (B) Mapping of the Sav interaction domain with Herc4. C-terminal fragments of Sav were used in co-immunoprecipitation experiments with Herc4. (C) Schematic representation of Herc4 and Sav truncations used in A and B.

To map the domains required for the interaction between Herc4 and Sav, we carried out co-IP experiments using fragments of either protein (Fig 7). Using this strategy, we were able to map the Herc4 interaction domain to the RLD domain as deleting the whole RLD abolishes binding between the two proteins. Deletion of RCC1-like repeats 1–3 had no effect on binding, neither did deletion of the entire HECT domain (Fig 7*A*). Thus, Herc4 binds Sav via RCC1-like domains 4–7.

Herc4 binds to the conserved C-terminus of the Sav protein (Fig 5*D*). To precisely define the region responsible for Sav/Herc4 binding, we analysed C-terminal fragments of Sav in co-IP experiments with Herc4 (Fig 7*B*). The domain responsible for the association of Herc4 to Sav can be narrowed down to the region stretching from the end of the WW domains to the beginning of the SARAH domain (aa 492–546), as deleting this domain abolishes binding between Herc4 and Sav (Fig 7*B*). Adding the SARAH domain (Sav∆N4) renders the interaction more robust, and elicits the appearance of a ladder of higher molecular species, which could indicate poly-ubiquitylation (Fig 7*B*).

### Antagonism between Herc4 and Hpo determines Sav stability

**Fig 8:**
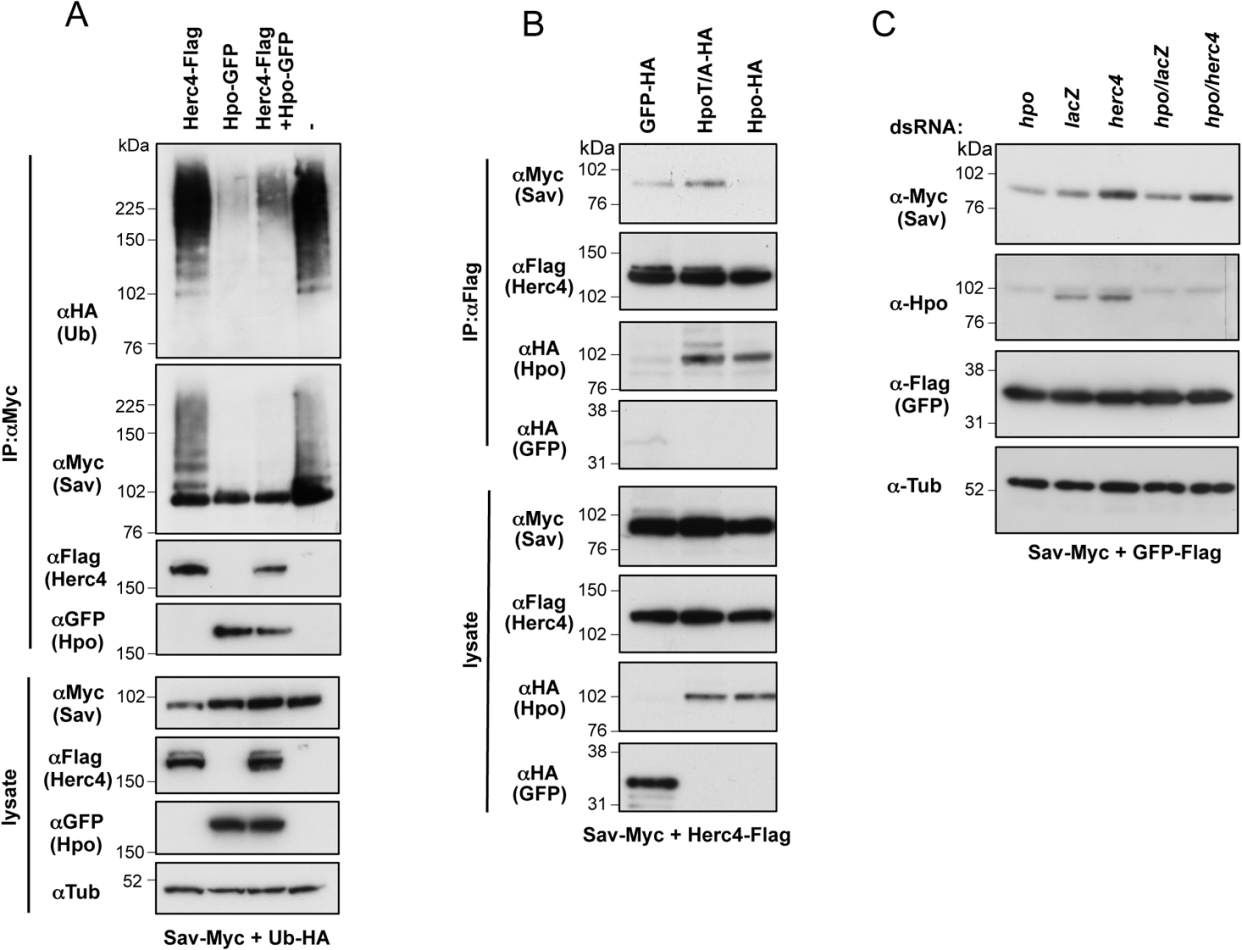
Hpo competes with Herc4 for Sav binding and antagonises Herc4-mediated ubiquitylation of Sav. **(**A) Hpo reduces Herc4-induced ubiquitylation of Sav. Sav ubiquitylation assay was performed using Myc-tagged Sav co-expressed with either Hpo, Herc4 or Hpo and Herc4 combined. Ubiquitylation assay was performed in the presence of 5mM NEM to block deubiquitylating enzyme activity. (B) Binding of Sav to Herc4 is prevented by active Hpo kinase (compare left with right lane), while kinase-dead Hpo has no effect (compare left and middle lanes). Herc4/Sav co-IP experiments were performed in the presence of a control protein (GFP), wild-type or kinase dead version of Hpo (HpoT195A). (C) *herc4* RNAi rescues the destabilizing effect of *hpo* RNAi on Sav protein levels. S2 cells expressing Myc-tagged Sav (and GFP-Flag as a control) were treated with the indicated combinations of dsRNA.

As both Herc4 and Hpo have a role in regulating Sav ubiquitylation and stability, we tested a possible functional interaction between the two proteins. Combined expression of Hpo and Herc4 resulted in reduced Herc4-induced Sav ubiquitylation (Fig 8*A*). We also noted that upon Hpo co-expression there was reduced association between Herc4 and Sav (Fig 8*A*). Similarly, Hpo reduced Sav levels in Herc4 immunoprecipitates (Fig 8*B*). Interestingly, the kinase dead version of Hpo (HpoT195A) did not affect Sav/Herc4 binding (Fig 8*B*), indicating that the effect of Hpo is phosphorylation-dependent. These results suggest that phosphorylation of Sav by Hpo prevents Herc4/Sav binding, leading to Sav stabilization. Depletion of *hpo* causes Sav to be destabilized (Fig 2*B*), suggesting that under those conditions Sav is no longer protected from Herc4-mediated degradation. Indeed, co-depletion of *herc4* and *hpo* restored Sav levels (Fig 8*C*). Thus, the interplay between Hpo and Herc4 determines Sav stability.

### *herc4* depletion prevents Sav destabilization caused by loss of apical Hpo pathway regulators

Recent reports have highlighted the importance of the cell cortex as an organising centre for Hpo pathway activity [44,47,55–58]. Several apically localized proteins, such as the Kibra/Expanded/Merlin (KEM) complex and the immunoglobulin super-family trans-membrane protein Echinoid (Ed), have been proposed to recruit Hpo/Sav to the cell membrane. In particular, Ed has been shown to bind Sav and prevent its degradation [55].

**Fig 9:**
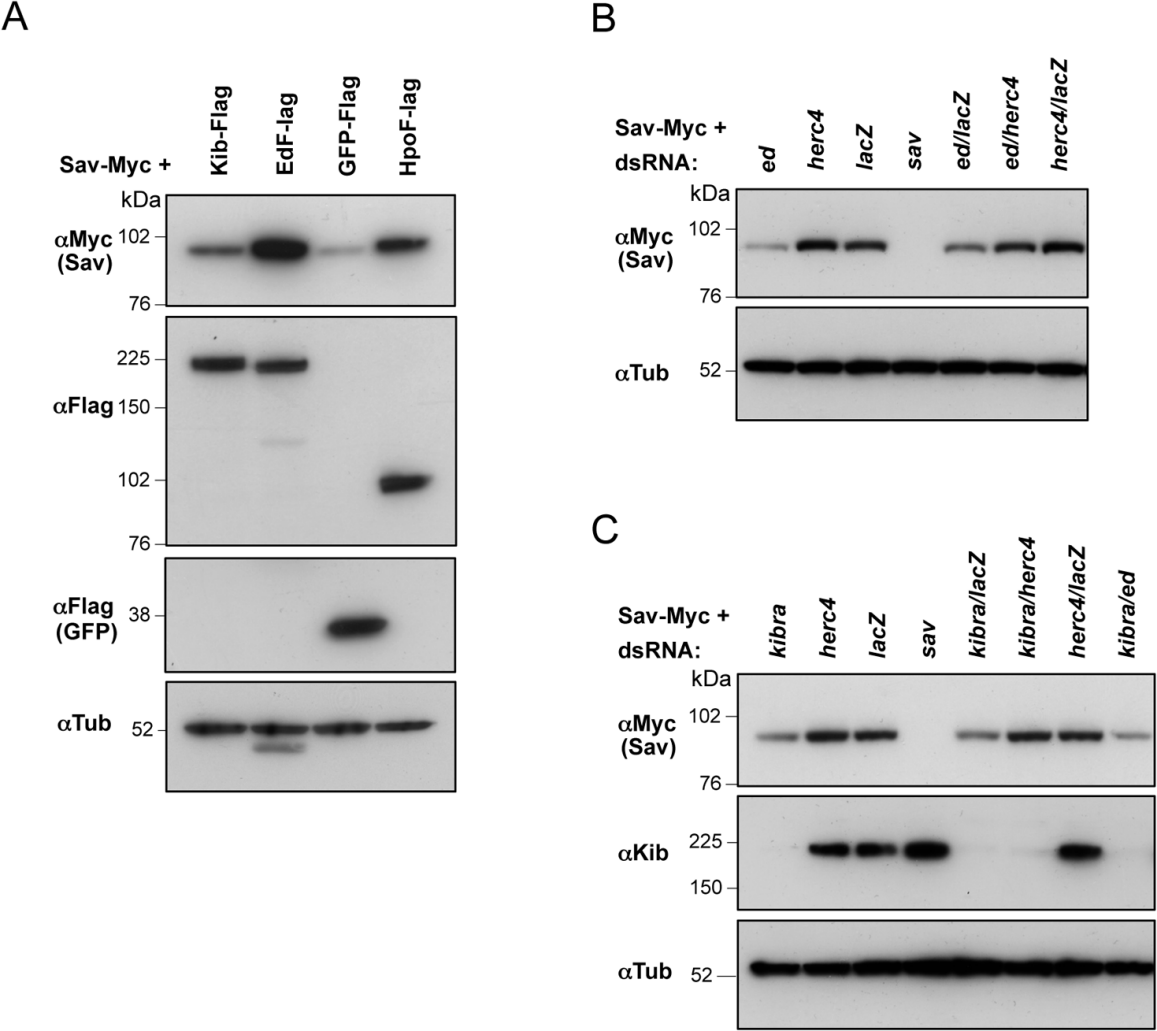
*herc4* depletion prevents the decrease in Sav protein levels elicited by loss of apical Hpo pathway regulators. **(**A) Co-expression of Ed and Kibra stabilizes Myc-tagged Sav. Hpo co-transfection is used as a positive control. (B) *herc4* RNAi rescues *ed* RNAi-induced reduction in Sav protein levels. (C) *herc4* RNAi rescues *kibra* RNAi-induced reduction in Sav protein levels. (A-C) S2 cells were transfected with Sav-Myc and the indicated plasmids, and treated with the indicated dsRNAs before lysis and western blot analysis.

Expression of Ed and Kibra in S2 cells stabilises co-transfected Sav (Fig 9*A* and [55]). Conversely, loss of either Kibra or Ed causes a marked reduction of Sav levels (Fig 9*B* and 9*C*), suggesting that loss of apical tethering of Sav makes it a target for ubiquitylation and degradation. To investigate this assumption further, we tested the effect of depleting *herc4* in this context. S2 cells were transfected with Sav-Myc and treated with dsRNA for *kibra* and *ed* in combination with *herc4* RNAi or *lacZ* RNAi as a control. *herc4* RNAi rescued the destabilising effect of *ed* and *kibra* RNAi (Fig 9*B* and 9*C*). Thus, when apical members of the Hpo pathway are depleted, Sav is released into the cytosol, where Herc4 promotes its ubiquitylation and subsequent degradation by the proteasome.

### Growth regulatory function of Herc4

**Fig 10:**
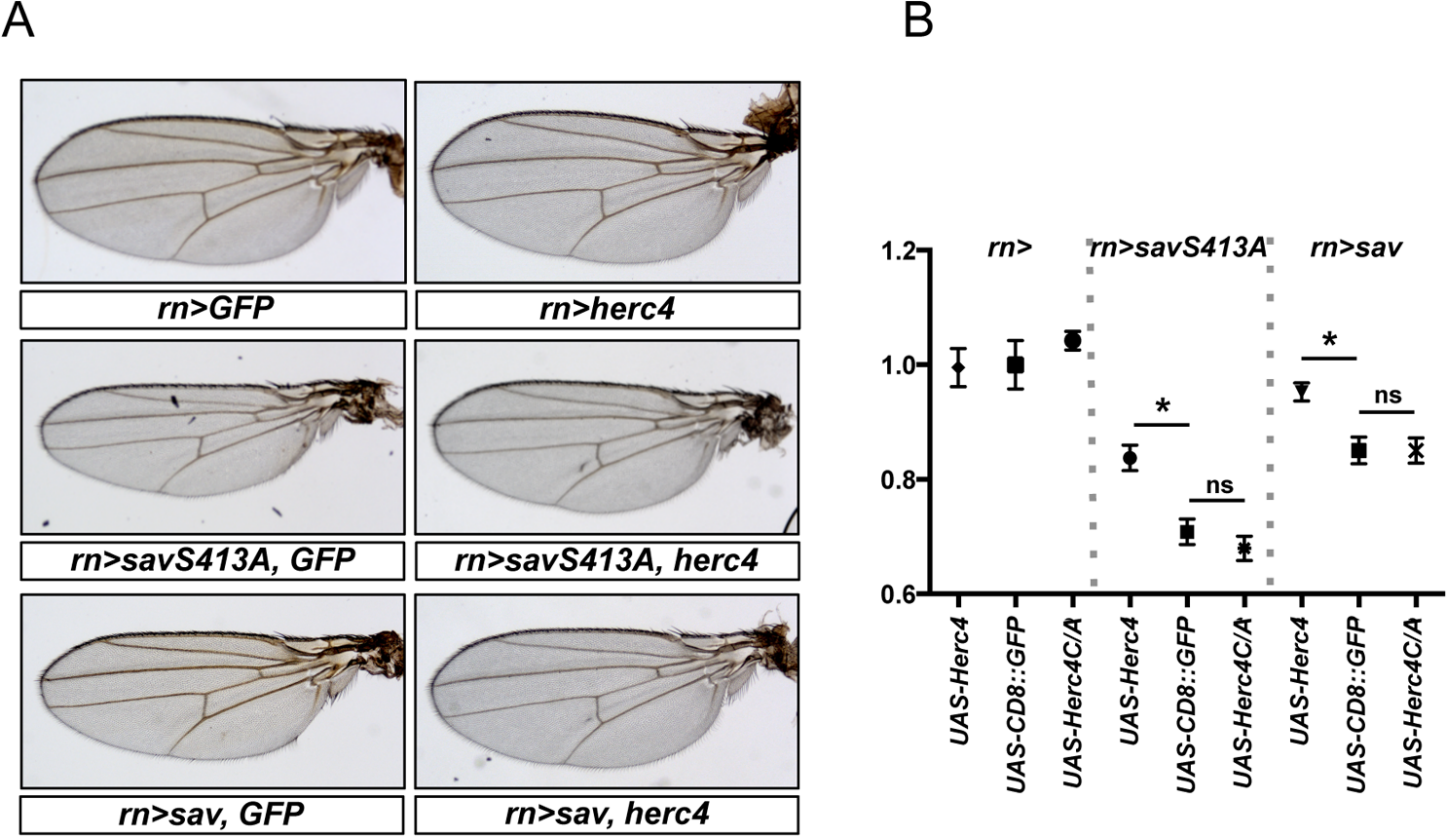
Growth regulatory function of Herc4. **(**A) Overexpression of Herc4 leads to a partial rescue of the Sav overexpression phenotype. Expression of *sav* (*rn>sav*) and *savS413A* (*rn>savS413A*) transgenes under the control of the *rotund* (*rn*) promoter causes tissue undergrowth and results in smaller wings. Both transgenes were combined with either *UAS-herc4*, *UAS-herc4C/A* or *UAS-CD8-GFP* to address the effect of Herc4. (B) Quantification of wing sizes. Shown are fold changes of average wing areas for the indicated genotypes compared to the control (*rn>UAS-CD8-GFP*), which was set as 1. Statistical significance assessed by Student’s t-test. * *p* < 0.0001. N.S. *p* > 0.05. n = 10–21. Error bars denote standard deviations.

One of the main effects of Hpo pathway disruption *in vivo* is an increase in tissue size. To investigate the impact of Herc4 on tissue growth control via Sav, we combined GAL4/UAS-driven Herc4 expression with transgenic lines expressing either wild type Sav or a version which is refractory to inhibition by the Salt-Inducible Kinases (Sik2/3) and therefore more active (Sav^S413A^) [59]. The transgenes were expressed under the control of the wing-specific driver *rotund-Gal4* (*rn-Gal4*). Overexpression of wild type Sav modestly reduced the size of the wing, while the Sav^S413A^ mutant had a stronger effect (Fig 10*A*). Expression of *UAS-herc4* was sufficient to partially suppress the undergrowth phenotype caused by Sav overexpression. This effect was dependent on the E3 ligase activity of Herc4, since overexpression of a HECT mutant version of Herc4 (*UAS-Herc4C/A*) had no effect on wing size (Fig 10*A* and 10*B*). Thus, Herc4 expression is able to suppress Sav growth-inhibitory function *in vivo*.

**Fig 11:**
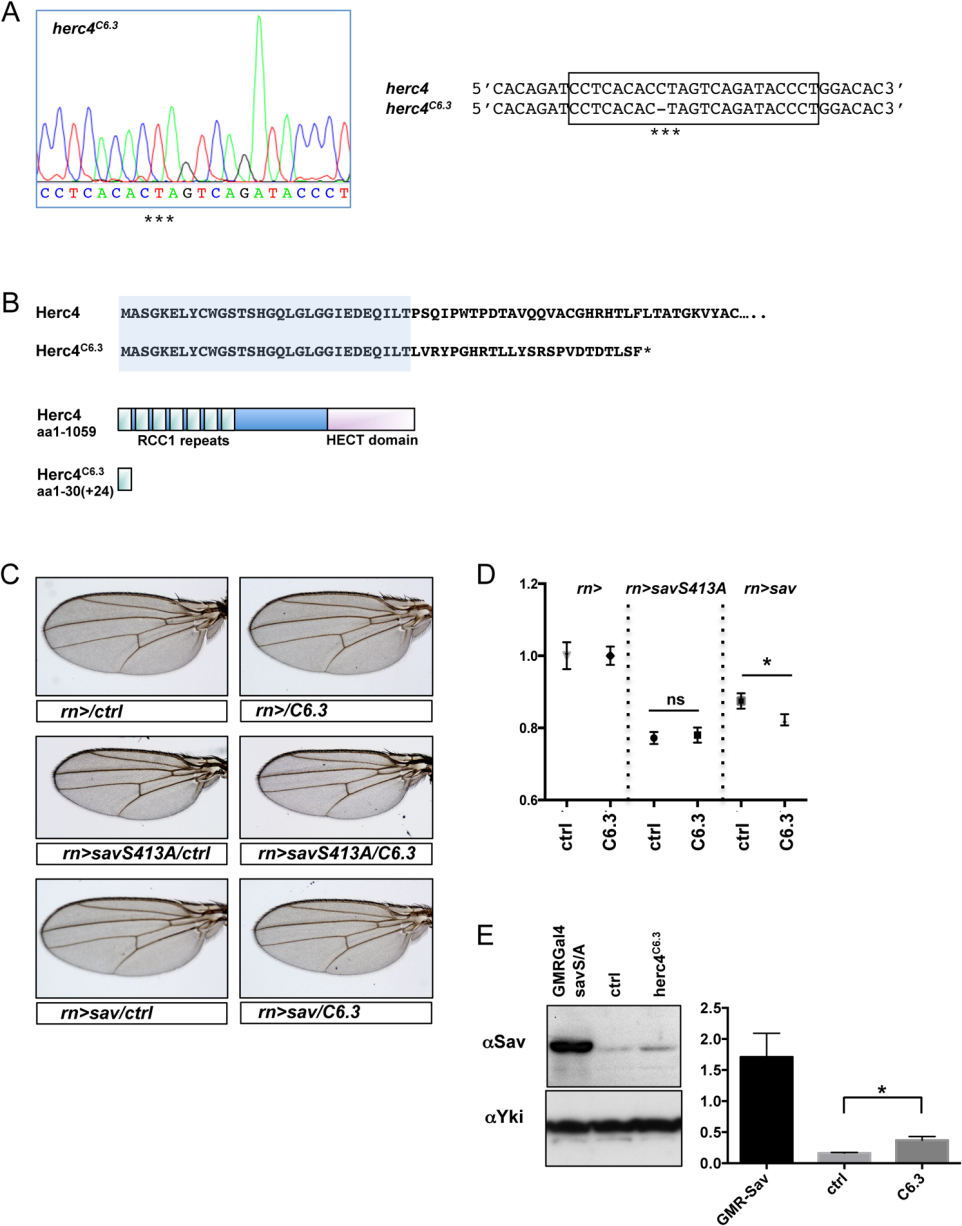
Generation and *in vivo* analysis of *herc4* ^*C6*.*3*^ mutant. **(**A) Sequence analysis of *herc4* CRISPR mutation. Sequence chromatogram of the sgRNA target sequence (boxed in the sequence alignment on the right) showing the loss of cytosine 92 (C92) in the *herc4* ^*C6*.*3*^ strain (marked by asterisk). (B) Diagram and sequence of the predicted protein resulting from frame-shift mutation. The loss of C92 results in a frame-shift mutation and a truncated protein of 30aa plus 24aa of out-of-frame sequence. (C) *herc4* ^*C6*.*3*^ enhances the Sav overexpression phenotype in the wing. Both *sav* (*rn>sav*) and *savS413A* (*rn>savS413A*) transgenes were combined with heterozygosity for either *herc4* ^*C6*.*3*^ or *herc4* ^*ctrl*^ and wings sizes were analysed. (D) Quantification of wing sizes. Shown are fold changes of average wing areas for the indicated genotypes compared to the control (*rn/ctrl*), which was set as 1. Statistical significance assessed by Student’s t-test. * *p* < 0.0001. N.S. *p* > 0.05. n = 10–21. Error bars denote standard deviations. (E) Levels of Sav protein in fly heads of *herc4* ^*C6*.*3*^, *herc4* ^*ctrl*^ and *GMR>savS413A* were analysed by Western blot. Bottom panel: quantification presented as ratio of Sav/Yki levels from three independent repeats. * *p* = 0.0385 with a Student’s t-test.

To study the effect of loss of Herc4 activity we generated a *herc4* mutant using CRISPR technology [49]. Using an sgRNA that targets the second exon of *herc4*, we generated a mutation, *herc4* ^*C6*.*3*^, carrying a one base-pair deletion at codon position C92 (cysteine 92) (Fig 11*A*). This mutation induces a frame-shift resulting in the production of a 30aa peptide (followed by 24aa of out-of-frame sequence), which lacks most of the Herc4 protein (Fig 11*B*). Homozygous *herc4* ^*C6*.*3*^ mutant animals were viable and fertile and had no apparent morphological abnormalities. This is not surprising, as modest overexpression of Sav, for instance in the eye under the *GMR* promoter, does not lead to a visible phenotype [5]. We therefore tested whether *herc4* ^*C6*.*3*^ could modify the undergrowth phenotype elicited by strong overexpression of Sav in the wing under the *rn-GAL4* driver (Fig 11*C* and 11*D*). Interestingly, loss of *herc4* ^*C6*.*3*^ does not modify the undergrowth induced by *savS413A*, which is already an activated form. By contrast, the *herc4* ^*C6*.*3*^ mutant significantly enhanced the undergrowth induced by wild type Sav overexpression.

To test whether loss of *herc4* affects Sav protein levels *in vivo*, we analysed Sav protein levels in fly heads by Western blot. Fig 11*E* shows that Sav protein levels were increased in *herc4* ^*C6*.*3*^ heads compared to control heads. Together, these results support a role for Herc4 as a Sav E3 ligase.

## Discussion

The scaffold protein Sav is a core component of the Hpo signalling pathway and its genetic inactivation is sufficient to increase tissue growth in flies and mice [5,6,41,43]. Sav dimerises with Hpo through their respective SARAH domains [7–11], and recruits Hpo to the plasma membrane, thereby promoting Wts phosphorylation [44]. In addition, Sav associates with the downstream kinase Wts via a WW domain/PPxY interaction [5]. Sav may therefore scaffold the Hpo/Wts core kinase cassette, thereby further strengthening Wts phosphorylation and activation. Indeed, mammalian Sav1/WW45 immunoprecipitates with endogenous Lats1 and Mst2 [21], and Sav strengthens the Hpo/Wts association in *Drosophila* tissue culture cells [59].

Sav levels appear to be tightly regulated by proteasomal degradation, and its association with Hpo/MST promotes both its phosphorylation and stabilisation ([7,9,45,46], this report). Using a point mutation in the ATP-binding pocket (Lysine to Arginine, K/R), we and others had observed that Hpo/MST kinase activity appeared dispensable for Sav stabilisation, opening the possibility for the involvement of another kinase [9,45]. However, since a version of Hpo carrying a mutation in the T-loop (T195A) and a truncation lacking the kinase domain both fail to phosphorylate and stabilise Sav (Fig 3), it seems most likely that Hpo kinase activity is in fact responsible for stabilising Sav, suggesting that the HpoK/R mutant has residual activity.

In order to shed light on the regulation of Sav stability, we performed a screen for Sav E3 ligases, leading us to identify Herc4 as a negative regulator of Sav (Fig 4). We show that *herc4* depletion reduces Sav ubiquitylation in cell culture (Fig 5) and that *herc4* mutants have elevated Sav levels in vivo (Fig 11). However, since the increase in Sav levels in *herc4* mutants are relatively modest, it is possible that other Sav ubiquitin ligases operate in parallel to Herc4. The Herc family is a poorly characterised group of six proteins containing a C-terminal HECT ubiquitin ligase domain and at least one N-terminal RLD [52]. Mammalian Herc1 and 2 are large (~500 KDa) proteins containing multiple RLDs as well as other domains (WD40, zinc fingers), while Herc3-6 have shorter N-termini containing one RLD domain. As a β-propeller structure, the RLD domain is likely to constitute a substrate-recruitment motif [53]. Indeed, we showed that Sav binding requires RCC1 repeats 4–7 (Fig 7). *Drosophila* has a single Herc1/2 ortholog (called Herc2) and a single Herc3-6 ortholog (CG9153, which we name Herc4 based on sequence homology). Herc4 mutant mice are viable but males display a spermatozoon maturation defect [60]. In addition, Herc4 was found to be upregulated in over half of a cohort of invasive ductal breast carcinoma [61]. It would therefore be interesting to examine Sav1/WW45 levels in these tumours, as well as assess their differentiation status, since recent work shows that Sav is required for terminal differentiation in the mouse breast epithelium [42].

In this report, we have also shown that the ability of Herc4 to degrade Sav is antagonised by Hpo (Fig 8). Furthermore, Hpo co-expression is able to displace Herc4 from Sav in a kinase domain-dependent manner. This could be due to phosphorylation by Hpo of the interaction surface on either Sav or Herc4. Our data are consistent with a model in which apical recruitment of Sav by proteins such as Ed and the KEM complex can stabilise Sav, perhaps by bringing it in close proximity to active Hpo at the plasma membrane. This would lead to stabilisation of Sav, promote Wts activation by Hpo and, ultimately, lead to Yki inhibition. We therefore propose that Hpo can strengthen the activation of the Hpo pathway core kinase cassette by phosphorylating its own adaptor Sav, thereby preserving it from Herc4-dependent degradation.

## Supporting Information

S1 Fig The Hect E3 ligases, Su(dx) and Nedd4, do not increase Sav ubiquitylation. Flag-tagged Herc4, Su(dx) and Nedd4 were expressed in S2 cells together with Sav-Myc and Ub-HA. After lysis, Sav was analysed for ubiquitylation.
(TIF)

S2 Fig RNAi library generation work plan. (TIF)

S3 Fig RNAi screen in S2R+ cells. (TIF)

S4 Fig Lysis and Western Blot analysis. (TIF)

## References

[1] Yu FX, Guan KL (2013) The Hippo pathway: regulators and regulations. Genes Dev 27: 355–371. 10.1101/gad.210773.112 23431053 PMC3589553

[2] Lin JI, Poon CL, Harvey KF (2013) The Hippo size control pathway—ever expanding. Sci Signal 6: pe4. 23354686 10.1126/scisignal.2003813

[3] Justice RW, Zilian O, Woods DF, Noll M, Bryant PJ (1995) The Drosophila tumor suppressor gene warts encodes a homolog of human myotonic dystrophy kinase and is required for the control of cell shape and proliferation. Genes Dev 9: 534–546. 7698644 10.1101/gad.9.5.534

[4] Xu T, Wang W, Zhang S, Stewart RA, Yu W (1995) Identifying tumor suppressors in genetic mosaics: the Drosophila lats gene encodes a putative protein kinase. Development 121: 1053–1063. 7743921 10.1242/dev.121.4.1053

[5] Tapon N, Harvey KF, Bell DW, Wahrer DC, Schiripo TA, Haber D, et al (2002) Salvador Promotes both cell cycle exit and apoptosis in Drosophila and is mutated in human cancer cell lines. Cell 110: 467–478. 12202036 10.1016/s0092-8674(02)00824-3

[6] Kango-Singh M, Nolo R, Tao C, Verstreken P, Hiesinger PR, Bellen HJ, et al (2002) Shar-pei mediates cell proliferation arrest during imaginal disc growth in Drosophila. Development 129: 5719–5730. 12421711 10.1242/dev.00168

[7] Wu S, Huang J, Dong J, Pan D (2003) Hippo encodes a Ste-20 family protein kinase that restricts cell proliferation and promotes apoptosis in conjunction with salvador and warts. Cell 114: 445–456. 12941273 10.1016/s0092-8674(03)00549-x

[8] Harvey KF, Pfleger CM, Hariharan IK (2003) The Drosophila Mst ortholog, hippo, restricts growth and cell proliferation and promotes apoptosis. Cell 114: 457–467. 12941274 10.1016/s0092-8674(03)00557-9

[9] Pantalacci S, Tapon N, Léopold P (2003) The Salvador partner Hippo promotes apoptosis and cell-cycle exit in Drosophila. Nat Cell Biol 5: 921–927. 14502295 10.1038/ncb1051

[10] Udan RS, Kango-Singh M, Nolo R, Tao C, Halder G (2003) Hippo promotes proliferation arrest and apoptosis in the Salvador/Warts pathway. Nat Cell Biol 5: 914–920. 14502294 10.1038/ncb1050

[11] Jia J, Zhang W, Wang B, Trinko R, Jiang J (2003) The Drosophila Ste20 family kinase dMST functions as a tumor suppressor by restricting cell proliferation and promoting apoptosis. Genes Dev 17: 2514–2519. 14561774 10.1101/gad.1134003 PMC218145

[12] Lai ZC (2005) Control of cell proliferation and apoptosis by mob as tumor suppressor, mats. Cell 120: 675–685. 15766530 10.1016/j.cell.2004.12.036

[13] Huang J, Wu S, Barrera J, Matthews K, Pan D (2005) The Hippo signaling pathway coordinately regulates cell proliferation and apoptosis by inactivating Yorkie, the Drosophila homolog of YAP. Cell 122: 421–434. 16096061 10.1016/j.cell.2005.06.007

[14] Dong J (2007) Elucidation of a universal size-control mechanism in Drosophila and mammals. Cell 130: 1120–1133. 17889654 10.1016/j.cell.2007.07.019 PMC2666353

[15] Oh H, Irvine KD (2008) In vivo regulation of Yorkie phosphorylation and localization. Development 135: 1081–1088. 10.1242/dev.015255 18256197 PMC2387210

[16] Wu S, Liu Y, Zheng Y, Dong J, Pan D (2008) The TEAD/TEF family protein scalloped mediates transcriptional output of the Hippo growth-regulatory pathway. Dev Cell 14: 388–398. 10.1016/j.devcel.2008.01.007 18258486

[17] Zhao B (2008) TEAD mediates YAP-dependent gene induction and growth control. Genes Dev 22: 1962–1971. 10.1101/gad.1664408 18579750 PMC2492741

[18] Thompson BJ, Cohen SM (2006) The Hippo pathway regulates the bantam microRNA to control cell proliferation and apoptosis in Drosophila. Cell 126: 767–774. 16923395 10.1016/j.cell.2006.07.013

[19] Nolo R, Morrison CM, Tao C, Zhang X, Halder G (2006) The bantammicroRNA is a target of the hippo tumor-suppressor pathway. Curr Biol 16: 1895–1904. 16949821 10.1016/j.cub.2006.08.057

[20] Neto-Silva RM, de Beco S, Johnston LA (2010) Evidence for a growth-stabilizing regulatory feedback mechanism between Myc and Yorkie, the Drosophila homolog of Yap. Dev Cell 19: 507–520. 10.1016/j.devcel.2010.09.009 20951343 PMC2965774

[21] Guo C, Tommasi S, Liu L, Yee JK, Dammann R, Pfeiffer GP (2007) RASSF1A is part of a complex similar to the Drosophila Hippo/Salvador/Lats tumor-suppressor network. Curr Biol 17: 700–705. 17379520 10.1016/j.cub.2007.02.055

[22] Matallanas D, Romano D, Yee K, Meissl K, Kucerova L, Piazzolla D, et al (2007) RASSF1A elicits apoptosis through an MST2 pathway directing proapoptotic transcription by the p73 tumor suppressor protein. Mol Cell 27: 962–975. 17889669 10.1016/j.molcel.2007.08.008 PMC2821687

[23] Zhang J, Smolen GA, Haber DA (2008) Negative regulation of YAP by LATS1 underscores evolutionary conservation of the Drosophila Hippo pathway. Cancer Res 68: 2789–2794. 10.1158/0008-5472.CAN-07-6205 18413746

[24] Zhao B, Wei X, Li W, Udan RS, Yang Q, Joungmok K, et al (2007) Inactivation of YAP oncoprotein by the Hippo pathway is involved in cell contact inhibition and tissue growth control. Genes Dev 21: 2747–2761. 17974916 10.1101/gad.1602907 PMC2045129

[25] Oka T, Mazack V, Sudol M (2008) Mst2 and Lats kinases regulate apoptotic function of Yes kinase-associated protein (YAP). J Biol Chem 283: 27534–27546. 10.1074/jbc.M804380200 18640976

[26] Moya IM, Halder G (2014) Discovering the Hippo pathway protein-protein interactome. Cell Res 24: 137–138. 10.1038/cr.2014.6 24418760 PMC3915912

[27] Genevet A, Tapon N (2011) The Hippo pathway and apico-basal cell polarity. Biochem J 436: 213–224. 10.1042/BJ20110217 21568941

[28] Yu FX (2012) Regulation of the Hippo–YAP pathway by G-protein-coupled receptor signaling. Cell 150: 780–791. 10.1016/j.cell.2012.06.037 22863277 PMC3433174

[29] Liu CY, Zha ZY, Zhou X, Zhang H, Huang W, Zhao D (2010) The hippo tumor pathway promotes TAZ degradation by phosphorylating a phosphodegron and recruiting the SCF{beta}-TrCP E3 ligase. J Biol Chem 285: 37159–37169. 10.1074/jbc.M110.152942 20858893 PMC2988322

[30] Zhao B, Li L, Tumaneng K, Wang CY, Guan KL (2010) A coordinated phosphorylation by Lats and CK1 regulates YAP stability through SCF(β-TRCP). Genes Dev 24: 72–85. 10.1101/gad.1843810 20048001 PMC2802193

[31] Song MS, Song SJ, Kim SJ, Nakayama K, Nakayama KI, Lim D-S (2008) Skp2 regulates the antiproliferative function of the tumor suppressor RASSF1A via ubiquitin-mediated degradation at the G1-S transition. Oncogene 27: 3176–3185. 18071316 10.1038/sj.onc.1210971

[32] Suryaraja R, Anitha M, Anbarasu K, Kumari G, Mahalingam S (2013) The E3 ubiquitin ligase Itch regulates tumor suppressor protein RASSF5/NORE1 stability in an acetylation-dependent manner. Cell Death Dis 4: e565 10.1038/cddis.2013.91 23538446 PMC3615736

[33] Lignitto L, Arcella A, Sepe M, Rinaldi L, Delle Donne R, Gallo A, et al (2013) Proteolysis of MOB1 by the ubiquitin ligase praja2 attenuates Hippo signalling and supports glioblastoma growth. Nat Commun 4: 1822 10.1038/ncomms2791 23652010 PMC3674242

[34] Salah Z, Melino G, Aqeilan RI (2011) Negative regulation of the Hippo pathway by E3 ubiquitin ligase ITCH is sufficient to promote tumorigenicity. Cancer Res 71: 2010–2020. 10.1158/0008-5472.CAN-10-3516 21212414

[35] Yeung B, Ho KC, Yang X (2013) WWP1 E3 ligase targets LATS1 for ubiquitin-mediated degradation in breast cancer cells. PLoS One 8: e61027 10.1371/journal.pone.0061027 23573293 PMC3616014

[36] Xiao L, Chen Y, Ji M, Dong J (2011) KIBRA regulates Hippo signaling activity via interactions with large tumor suppressor kinases. J Biol Chem 286: 7788–7796. 10.1074/jbc.M110.173468 21233212 PMC3048666

[37] Scheel H, Hofmann K (2003) A novel interaction motif, SARAH, connects three classes of tumor suppressor. Curr Biol 13: R899–900. 14654011 10.1016/j.cub.2003.11.007

[38] Praskova M, Khoklatchev A, Ortiz-Vega S, Avruch J (2004) Regulation of the MST1 kinase by autophosphorylation, by the growth inhibitory proteins, RASSF1 and NORE1, and by Ras. Biochem J 381: 453–462. 15109305 10.1042/BJ20040025 PMC1133852

[39] Ni L, Li S, Yu J, Min J, Brautigam CA, Tomchick DR, et al (2013) Structural basis for autoactivation of human Mst2 kinase and its regulation by RASSF5. Structure 21: 1757–1768. 10.1016/j.str.2013.07.008 23972470 PMC3797246

[40] Deng Y, Matsui Y, Zhang Y, Lai ZC (2013) Hippo activation through homodimerization and membrane association for growth inhibition and organ size control. Dev Biol 375: 152–159. 10.1016/j.ydbio.2012.12.017 23298890

[41] Lee KP, Lee JH, Kim TS, Kim TH, Park HD, Byun JS, et al (2010) The Hippo-Salvador pathway restrains hepatic oval cell proliferation, liver size, and liver tumorigenesis. Proc Natl Acad Sci U S A 107: 8248–8253. 10.1073/pnas.0912203107 20404163 PMC2889558

[42] Chen Q, Zhang N, Gray RS, Li H, Ewald AJ, Zahnow CA, et al (2014) A temporal requirement for Hippo signaling in mammary gland differentiation, growth, and tumorigenesis. Genes Dev 28: 432–437. 10.1101/gad.233676.113 24589775 PMC3950341

[43] Lee JH, Kim TS, Yang TH, Koo BK, Oh SP, Lee K-P, et al (2008) A crucial role of WW45 in developing epithelial tissues in the mouse. EMBO J 27: 1231–1242. 10.1038/emboj.2008.63 18369314 PMC2367404

[44] Yin F, Yu J, Zheng Y, Chen Q, Zhang N, Pan D (2013) Spatial organization of Hippo signaling at the plasma membrane mediated by the tumor suppressor Merlin/NF2. Cell 154: 1342–1355. 10.1016/j.cell.2013.08.025 24012335 PMC3835333

[45] Callus BA, Verhagen AM, Vaux DL (2006) Association of mammalian sterile twenty kinases, Mst1 and Mst2, with hSalvador via C-terminal coiled-coil domains, leads to its stabilization and phosphorylation. FEBS J 273: 4264–4276. 16930133 10.1111/j.1742-4658.2006.05427.x

[46] Wu D, Wu S (2013) A conserved serine residue regulates the stability of Drosophila Salvador and human WW domain-containing adaptor 45 through proteasomal degradation. Biochem Biophys Res Commun 433: 538–541. 10.1016/j.bbrc.2013.03.023 23524264

[47] Genevet A, Wehr MC, Brain R, Thompson BJ, Tapon N (2010) Kibra is aregulator of the Salvador/Warts/Hippo signaling network. Dev Cell 18: 300–308. 10.1016/j.devcel.2009.12.011 20159599 PMC2845807

[48] Polesello C, Huelsmann S, Brown NH, Tapon N (2006) The Drosophila RASSF homolog antagonizes the hippo pathway. Curr Biol 16: 2459–2465. 17174922 10.1016/j.cub.2006.10.060 PMC1828611

[49] Port F, Chen HM, Lee T, Bullock SL (2014) Optimized CRISPR/Cas tools for efficient germline and somatic genome engineering in Drosophila. Proc Natl Acad Sci U S A 111: E2967–2976. 10.1073/pnas.1405500111 25002478 PMC4115528

[50] Nagy V, Dikic I (2010) Ubiquitin ligase complexes: from substrate selectivity to conjugational specificity. Biol Chem 391: 163–169. 10.1515/BC.2010.021 20030582

[51] Park BH, Lee YH (2011) Phosphorylation of SAV1 by mammalian ste20-like kinase promotes cell death. BMB Rep 44: 584–589. 21944251 10.5483/bmbrep.2011.44.9.584

[52] Hochrainer K, Mayer H, Baranyi U, Binder B, Lipp J, Kroismayr R (2005) The human HERC family of ubiquitin ligases: novel members, genomic organization, expression profiling, and evolutionary aspects. Genomics 85: 153–164. 15676274 10.1016/j.ygeno.2004.10.006

[53] Stevens TJ, Paoli M (2008) RCC1-like repeat proteins: a pangenomic, structurally diverse new superfamily of beta-propeller domains. Proteins 70: 378–387. 17680689 10.1002/prot.21521

[54] Scheffner M, Kumar S (2014) Mammalian HECT ubiquitin-protein ligases: biological and pathophysiological aspects. Biochim Biophys Acta 1843: 61–74. 10.1016/j.bbamcr.2013.03.024 23545411

[55] Yue T, Tian A, Jiang J (2012) The cell adhesion molecule echinoid functions as a tumor suppressor and upstream regulator of the Hippo signaling pathway. Dev Cell 22: 255–267. 10.1016/j.devcel.2011.12.011 22280890 PMC3288783

[56] Ho LL, Wei X, Shimizu T, Lai ZC (2010) Mob as tumor suppressor is activated at the cell membrane to control tissue growth and organ size in Drosophila. Dev Biol 337: 274–283. 10.1016/j.ydbio.2009.10.042 19913529

[57] Yu J, Zheng Y, Dong J, Klusza S, Deng WM, Pan D (2010) Kibra functions as a tumor suppressor protein that regulates Hippo signaling in conjunction with Merlin and Expanded. Dev Cell 18: 288–299. 10.1016/j.devcel.2009.12.012 20159598 PMC2858562

[58] Baumgartner R, Poernbacher I, Buser N, Hafen E, Stocker H (2010) The WWdomain protein Kibra acts upstream of Hippo in Drosophila. Dev Cell 18: 309–316. 10.1016/j.devcel.2009.12.013 20159600

[59] Wehr MC, Holder MV, Gailite I, Saunders RE, Maile TM, Ciirdaeva E, et al (2013) Salt-inducible kinases regulate growth through the Hippo signalling pathway in Drosophila. Nat Cell Biol 15: 61–71. 23263283 10.1038/ncb2658 PMC3749438

[60] Rodriguez CI, Stewart CL (2007) Disruption of the ubiquitin ligase HERC4 causes defects in spermatozoon maturation and impaired fertility. Dev Biol 312: 501–508. 17967448 10.1016/j.ydbio.2007.09.053

[61] Zhou H, Shi R, Wei M, Zheng WL, Zhou JY, Ma W-L (2013) The expression and clinical significance of HERC4 in breast cancer. Cancer Cell Int 13: 113 10.1186/1475-2867-13-113 24225229 PMC3832903

