# Supplementary figures and images for: Hippo Stabilises Its Adaptor Salvador by Antagonising the HECT Ubiquitin Ligase Herc4
Source: PLoS One. 2015 Jun 30;10(6):e0131113. doi: 10.1371/journal.pone.0131113 (PMC4488328; doi:10.1371/journal.pone.0131113)

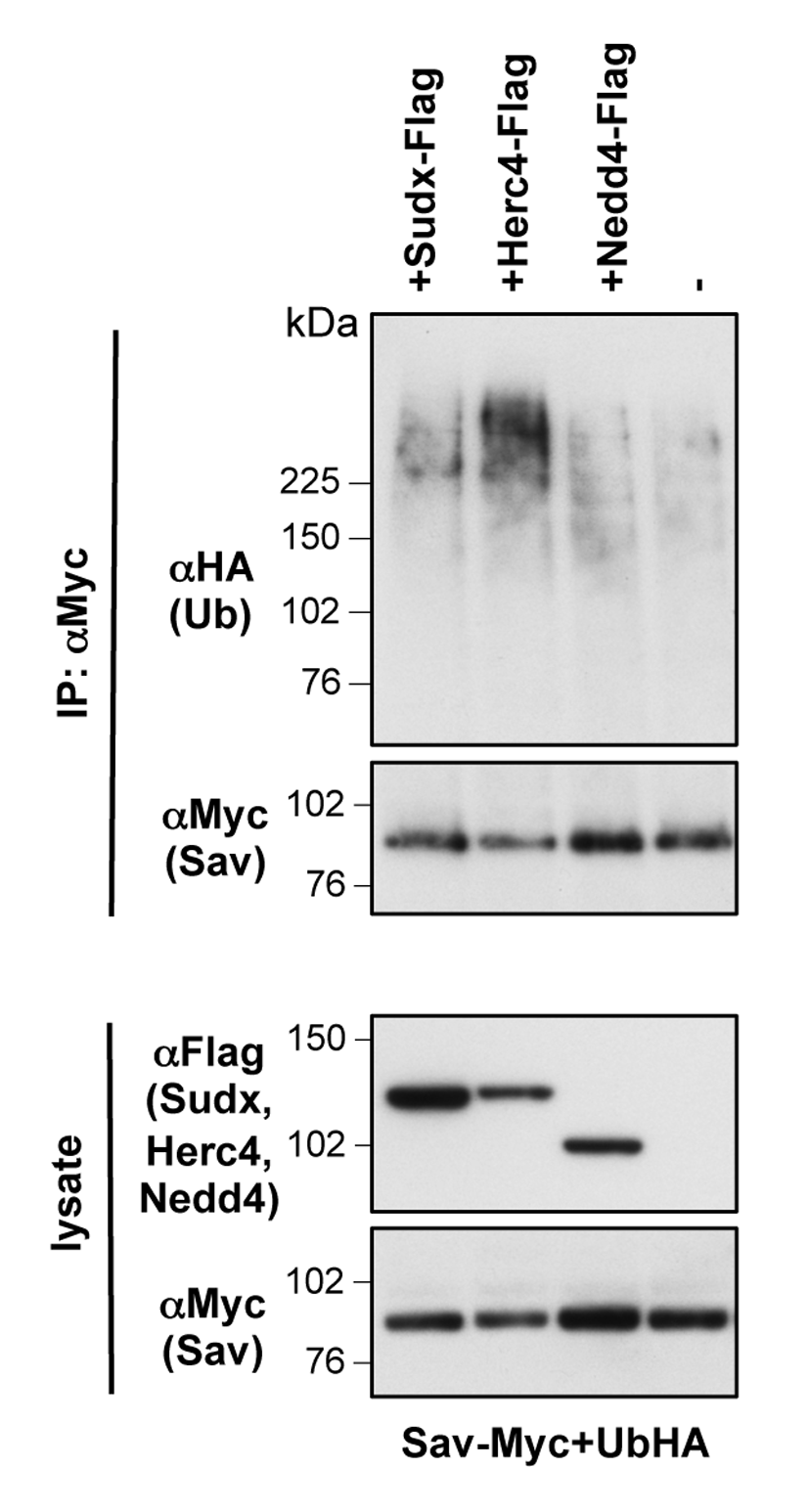

Supplement: S1 Fig — Flag-tagged Herc4, Su(dx) and Nedd4 were expressed in S2 cells together with Sav-Myc and Ub-HA. After lysis, Sav was analysed for ubiquitylation. (TIF) [file pone.0131113.s001.tif]

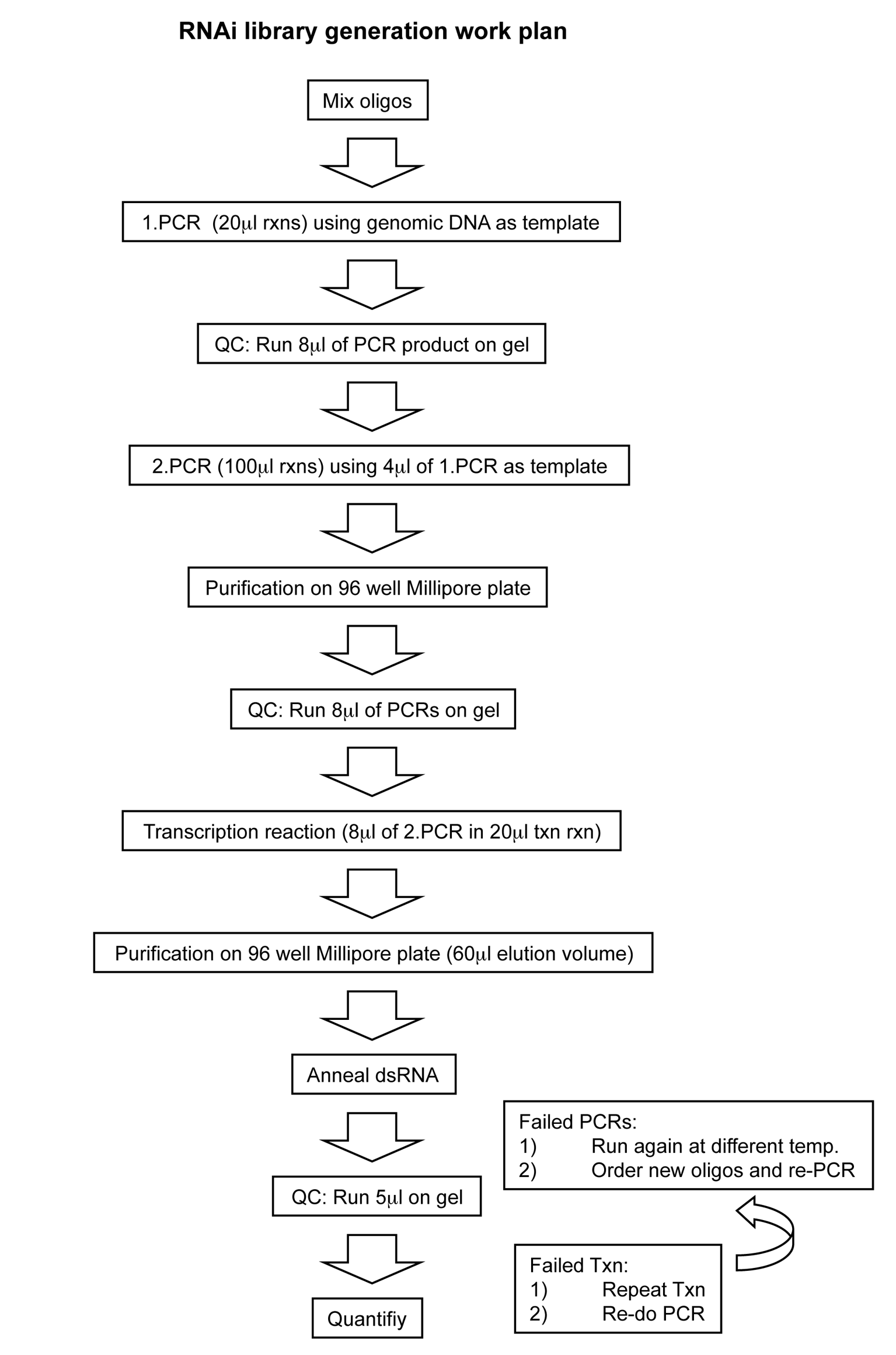

Supplement: S2 Fig — (TIF) [file pone.0131113.s002.tif]

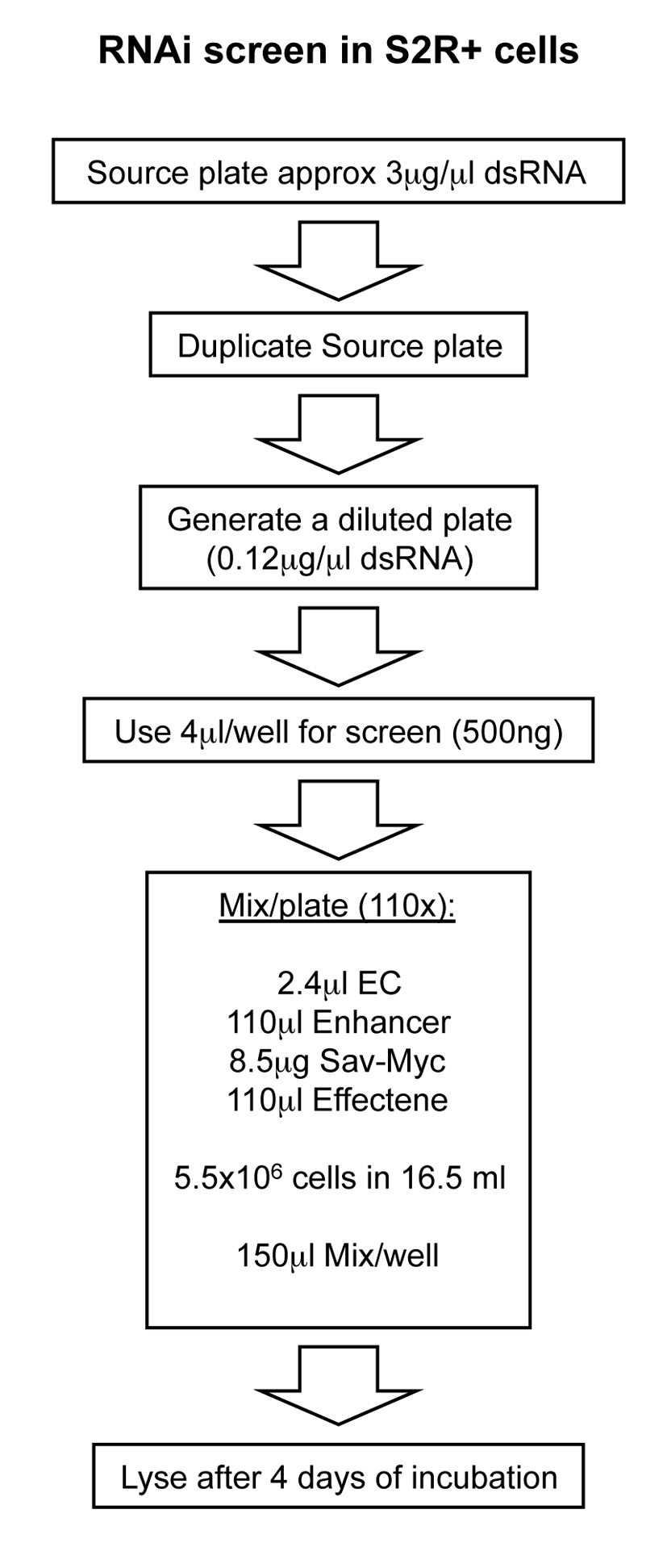

Supplement: S3 Fig — (TIF) [file pone.0131113.s003.tif]

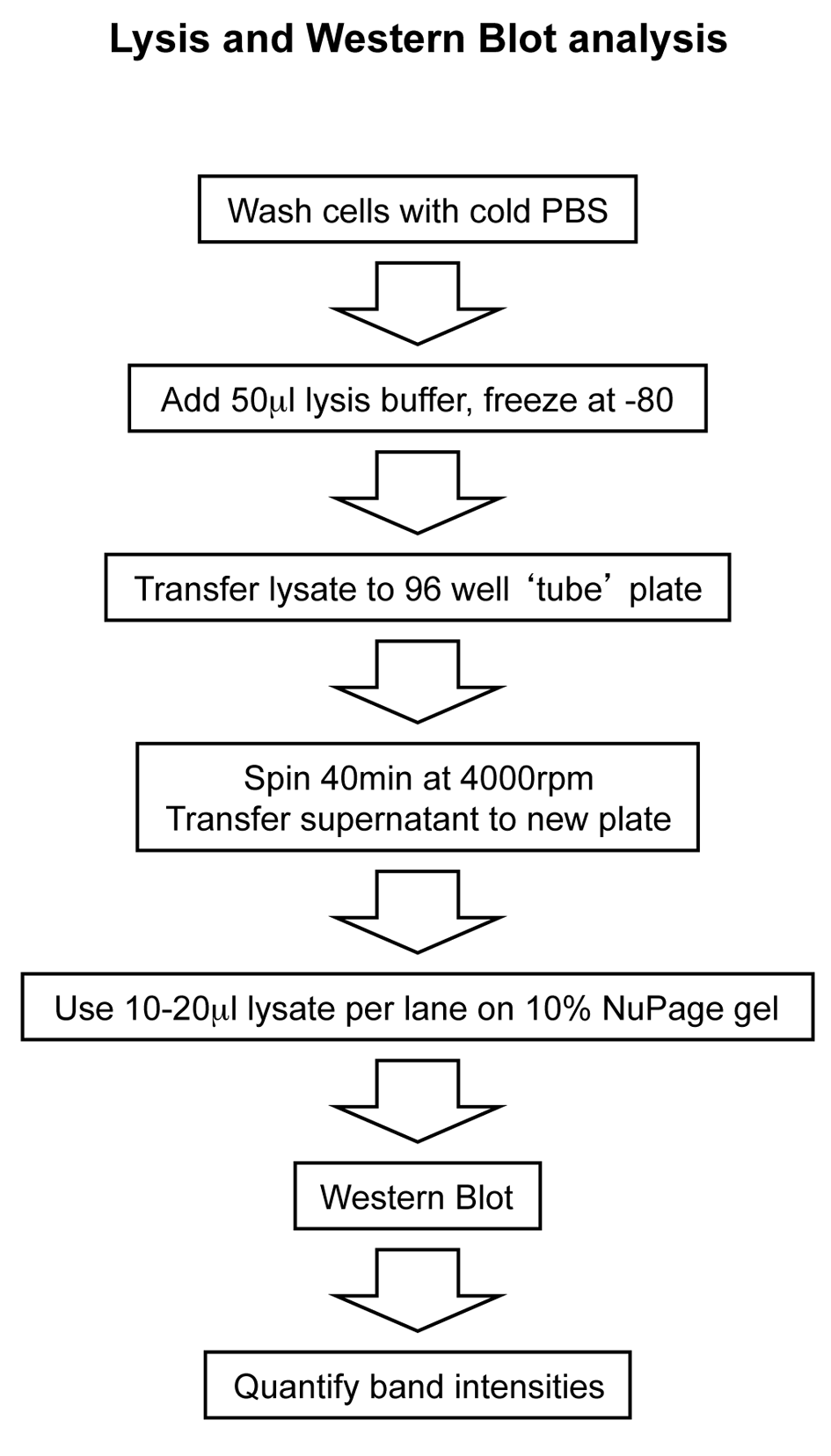

Supplement: S4 Fig — (TIF) [file pone.0131113.s004.tif]
